# Asymmetric Total Syntheses of Both Enantiomers of Plymuthipyranone B and Its Unnatural Analogues: Evaluation of *anti*-MRSA Activity and Its Chiral Discrimination

**DOI:** 10.3390/ph14090938

**Published:** 2021-09-19

**Authors:** Mizuki Moriyama, Xiaoxi Liu, Yuki Enoki, Kazuaki Matsumoto, Yoo Tanabe

**Affiliations:** 1 Department of Chemistry, School of Science and Technology, Kwansei Gakuin University, 2-1 Gakuen, Sanda 669-1337, Japan; ixu18409@kwansei.ac.jp; 2 Division of Pharmacodynamics, Faculty of Pharmacy, Keio University, 1-5-30 Shibakoen, Minato-ku, Tokyo 105-8512, Japan; aganeiliu@gmail.com (X.L.); enoki-yk@pha.keio.ac.jp (Y.E.); matsumoto-kz@pha.keio.ac.jp (K.M.)

**Keywords:** *anti*-MRSA activity, asymmetric total syntheses, enantiomers, chiral discrimination, plymuthipyranone B

## Abstract

Chiral total syntheses of both enantiomers of the *anti*-MRSA active plymuthipyranone B and all of the both enantiomers of three unnatural and synthetic analogues were performed. These two pairs of four chiral compounds are composed of the same 3-acyl-5,6-dihydro-2*H*-pyran-2-one structure. The starting synthetic step utilized a privileged asymmetric Mukaiyama aldol addition using Ti(O*i*Pr)_4_/(*S*)-BINOL or Ti(O*i*Pr)_4_/(*R*)-BINOL catalysis to afford the corresponding (*R*)- and (*S*)-δ-hydroxy-β-ketoesters, respectively, with highly enantiomeric excess (>98%). Conventional lactone formation and successive EDCI-mediated *C*-acylation produced the desired products, (*R*)- and (*S*)-plymuthipyranones B and three (*R*)- and (*S*)- synthetic analogues, with an overall yield of 42–56% with a highly enantiomeric excess (95–99%). A bioassay of the *anti*-MRSA activity against ATCC 43300 and 33591 revealed that (i) the MICs of the synthetic analogues against ATCC 43300 and ATCC 33591 were between 2 and 16 and 4 and 16 μg/mL, respectively, and those of vancomycin (reference) were 1 μg/mL. (ii) The natural (*S*)-plymuthipyranone B exhibited significantly higher activity than the unnatural (*R*)-antipode against both AACCs. (iii) The natural (*R*)-plymuthipyranone B and (*R*)-undecyl synthetic analogue at the C6 position exhibited the highest activity. The present work is the first investigation of the SAR between chiral *R* and *S* forms of this chemical class.

## 1. Introduction

**Figure 1:**
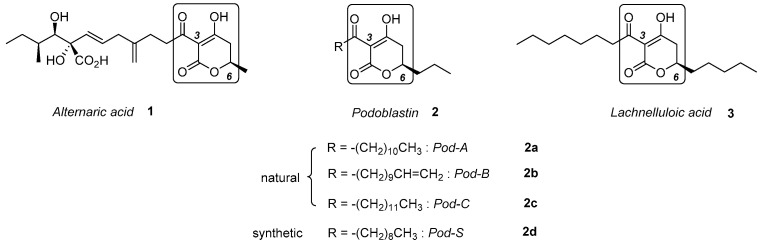
Representative natural products (**1**, **2a**–**c**, **3**) and a synthetic analogue (**2d**) containing the 3-acyl-5,6-dihydro-2*H*-pyran-2-one structure.

The chiral discrimination of bioactivity between enantiomers has occupied a central position in modern research and the development of pharmaceuticals and agrochemicals. 3-Acyl-5,6-dihydro-2*H*-pyran-2-ones are unique heterocyclic molecules with a tricarbonyl moiety at the C(3)-position and an asymmetric center at the C(6)-position [1]. Several natural products contain these compounds, as depicted in Figure 1.

Alternaric acid (**1**) with three contiguous stereocenters and non-conjugated dienes is the most representative phytotoxic and antifungal natural product isolated from *Alternaria solani* [2]. The distinctive and exquisite structure of **1** renders this compound a worthy synthetic target. The total or formal chiral syntheses of **1** have been achieved by the following several groups: (i) the first total synthesis started from (*S*)-methylbutanol and methyl (*R*)-hydroxybutanoate (Ichihara’s group) [3,4], (ii) formal synthesis utilizing Ru-catalyzed alder ene type reactions (Trost’s group) [5], (iii) formal synthesis utilizing a silyl glyoxylate three-component-coupling method (Johnson and Slade) [6] and (iv) total synthesis utilizing asymmetric Ti-Claisen condensation by our group [7,8].

Antifungal active (*R*)-podoblastins A–C (**2a**–**2c**) and S (**2d**) against rice-blast disease were isolated from *Podophyllum peltatum* L. [9] and synthesized as racemic forms utilizing a Fries-type acyl group rearrangement [10] and 1,3-dipolar cycloaddition [11]. A chiral pool total synthesis of (*R*)-podoblastins S (**2d**) starting from (*S*)-glycelaldehyde acetonide was reported [12]. A related antifungal antagonistic active (*R*)-lachnelluloic acid (**3**) against Dutch elm disease was isolated from *Lachnellula fuscosanguinea* (Rehm) Dennis, and its racemic form was synthesized [13]. Later, a formal synthesis of **3** was reported [14]. Recently, the chiral total syntheses of (*R*)-podoblastin S (**2d**) and (*R*)-lachnelluloic acid were performed utilizing catalytic asymmetric Mukaiyama aldol reactions [15]. 

**Figure 2:**
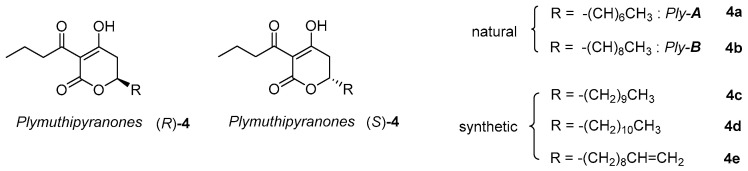
Natural plymuthipyranone A, B (**4a**, **4b**) and synthetic analogues (**4c**–**4e**) containing the 3-acyl-5,6-dihydro-2*H*-pyran-2-one structure.

Notably, despite these extensive studies, chiral discrimination studies of C(6*R*) and anti-podal C(6*S*) isomers have not yet been performed, simply due to the lack of an accessible method for synthesizing the 6(*S*) enantiomer, i.e., syntheses have been limited to C(6*R*) isomers. Very recently, Broberg’s group disclosed that plymuthipyranones A (**4a**) and B (**4b**), having a 3-butanoyl-5,6-dihydro-2*H*-pyran-2-one structure, which were isolated from *Serratia plymuthica strain* MF371-2, exhibited highly potent activity against Gram-positive *Staphylococcus aureus* LMG 15975 (MRSA) [16] (Figure 2). They reported that the absolute configuration of plymuthipyranones A and B is *R* [16], according to the analogous optical rotation minus value [9,15]. They reported that racemic plymuthipyranones B (**4b**) was more potent than racemic A (**4a**), based on the MIC (minimal inhibition concentration) value. In addition, the racemic synthetic analogue **4d** exhibited the highest *anti*-MRSA activity among **4a**–**4d** with variable C(6)-side chains [16,17]. 

Consistent with our continuing interest in chiral discrimination studies between enantiomers and diastereomers [18,19,20,21,22,23], a major topic in pharmaceutical and agrochemical research, we envisaged the chiral total syntheses of three sets of plymuthipyranones to evaluate the *anti*-MRSA activity of natural plymuthipyranone B (more active than A), synthetic analogues **4c**, **4d** and novel synthetic analogue **4e**. This study is closely related to our total synthetic studies of a 3-acyl-5,6-dihydro-2*H*-pyran-2-one series [7,10,15] and relevant 4-methoxy derivatives (all four stereoisomers of pestalotin) [24]. 

## 2. Results and Discussion

### 2.1. Synthesis of Three Sets of Natural Plymuthipyranone B and Two Sets of Synthetic Analogues

Our synthesis commenced with the privileged asymmetric Mukaiyama aldol addition using Ti(O*i*Pr)_4_/(*S*)-BINOL catalysis originally developed by Soriente and Scettri’s group [25,26,27,28], which consistently produced the (*R*)-aldol adducts. In addition, (*S*)-aldol adducts can be obtained in a similar and stereocomplementary manner by switching the chiral catalysis from (*S*)-BINOL to (*R*)-BINOL.

**Scheme 1:**
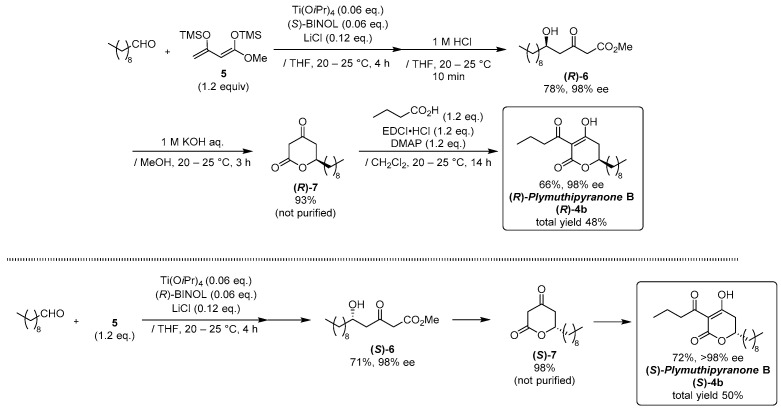
Asymmetric total synthesis of (*R*)- and (*S*)-plymuthipyranones B.

The reaction of 1,3-bis(trimethylsiloxy)diene (Chan’s diene) **5** [29,30,31] with decanal afforded (*R*)-δ-hydroxy-β-ketoester **6** with a 78% yield with an excellent 98% ee (Scheme 1). The conventional KOH-hydrolysis of (*R*)-**6** and the subsequent acid-catalyzed lactone formation afforded the desired (*R*)-5,6-dihydro-2*H*-pyran-2-one (*R*)-**7** with a 93% yield. For the *C*-acylation step, we adopted a mild and direct method utilizing an EDCI reagent [3,4] rather than an indirect *O*-acylation and successive Fries-type acyl group rearrangement [10,16]. Thus, the final EDCI-mediated *C*-acylation of (*R*)-**7** with butanoic acid furnished (*R*)-plymuthipyranone B [(*R*)-**4b**] with a 66% yield with 95% ee (HPLC analysis, SI). In a similar procedure, (*S*)-plymuthipyranone B [(*S*)-**4b**] was synthesized using Ti(O*i*Pr)_4_/anti-podal (*R*)-BINOL catalysis. Consequently, the present total syntheses of both enantiomers of plymuthipyranone B were performed in a total of five steps, achieving an overall yield of 48–50% with excellent enantioselectivity (95–97% ee).

Eventually, (*R*)- and (*S*)-stereocomplementary syntheses were performed by only switching (*S*)- and (*R*)-BINOL catalysts, respectively, both of which are commercially available with nearly the same prices.

**Scheme 2:**
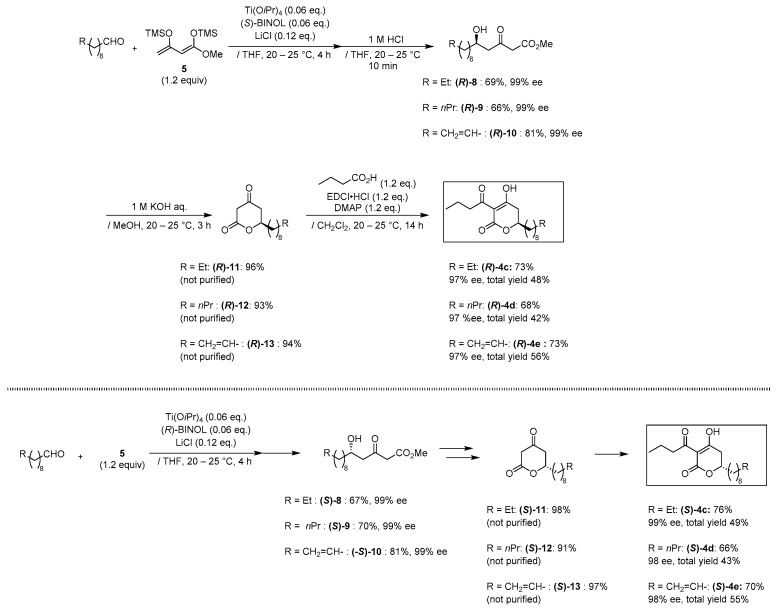
Asymmetric synthesis of (*R*)- and (*S*)-synthetic analogues of plymuthipyranones B.

We next turned our attention to the syntheses of the two enantiomer sets of unnatural (synthetic) analogues of (*R*)-**4c** and (*S*)-**4c**, (*R*)-**4d** and (*S*)-**4d**, respectively (Scheme 2). A racemic compound of **4d** exhibited the highest *anti*-MRSA activity among a series of these compounds [16]. Novel analogues (*R*)-, (*S*)-**4d** containing a terminal double bond in the substituent at the C(6)-position were selected as candidates, because (i) podoblastin B with a similar terminal double bond exhibited higher antifungal activity than podoblastins A and C with simple alkyl groups and (ii) starting 10-undecenal was commercially available. 

The syntheses of all the six target compounds were implemented similarly to the synthesis of plymuthipyranones B mentioned above. In all the cases, asymmetric Mukaiyama aldol addition using diene **5** to undecanal, 10-undecenal and dodecanal afforded (*R*)-**8**, (*R*)-**9**, (*R*)-**10**, (*S*)-**8**, (*S*)-**9** and (*S*)-**10** with a 66–81% yield with excellent enantioselectivity (99% ee). The subsequent KOH-hydrolysis and HCl treatment produced the corresponding (*R*)-5,6-dihydro-2*H*-pyran-2-ones (*R*)-**11**, (*R*)-**12**, (*R*)-**13**, (*S*)-**11**, (*S*)-**12** and (*S*)-**13**. The final *C*-acylations of (*R*)-**11**, (*R*)-**12**, (*R*)-**13**, (*S*)-**11**, (*S*)-**12** and (*S*)-**13** using the EDCI reagent furnished the target 4-acylated products (*R*)-**4c**, (*R*)-**4d**, (*R*)-**4e**, (*S*)-**4c,** (*S*)-**4d** and (*S*)-**4e**, respectively, with an acceptable overall yield (42–56%) with an excellent enantioselectivity (97–99% ee). 

### 2.2. Antibacterial Evaluation against MRSA

The stereostructure-activity relationships between all the enantiomers of plymuthipyranone B (**4b**) and synthetic analogues **4c**, **4d**, **4e** were assayed using two American Type Culture Collection (ATCC) cell lines (43300 and 33591) on the basis of their minimal inhibitory concentration (MIC) values against MRSA (Table 1). Broberg’s group reported that the *anti*-MRSA activity order was **4d** > **4c** > plymuthipyranone B (**4b**) > **4e** as “the racemic forms” [16]. 

The salient features are as follows: (i) The MICs of the synthetic analogues against ATCC 43300 and ATCC 33591 were between 2 and 16 and 4 and 16 μg/mL, respectively, and those of vancomycin (reference) were 1 μg/mL. (ii) As expected, natural plymuthipyranone B (*S*)-**4b** exhibited significantly higher activity than the unnatural antipode (*R*)-**4b** against both ATCCs. (iii) In clear contrast, reverse antipodal (*S*)-**4c** exhibited higher activity than (*R*)-**4c** against both ATCCs. (iv) Regarding the most active analogue **4d** (the racemic form), however, the activity was (*S*)-**4d** = (*R*)-**4d** against ATCC 3300 and (*S*)-**4d** > (*R*)-**4d** against ATCC 33591. (v) These results are in reasonable accordance with the reported data of racemates **4b**, **4c** and **4d**. (vi) (*R*)-**4e** and (*S*)-**4e** possessing a terminal double bond were quite less reactive in contrast to the SAR of podoblastins [15]. (vii) The MICs of the most active (*R*)-**4c** and (*S*)-**4d** were approximately half that of vancomycin. 

Among various biologically active 3-acyl-5,6-dihydro-2*H*-pyran-2-one compounds, this is the first investigation of the stereostructure-activity relationship (SAR) between chiral *R* and *S* forms. Eventually, the subtle changes of the chain length at the C6-position significantly influenced the inherent activity and chiral discrimination. On the other hand, the following SAR studies remain: (i) variation of 3-acyl substituent and (ii) isosterism for other related heterocycles such as 2*H*-pyrones, piperidine, etc. 

## 3. Materials and Methods 

### 3.1. Synthesis

All reactions were carried out in oven-dried glassware under an argon atmosphere. Flash column chromatography was performed with silica gel 60 (230–400 mesh ASTM, Merck, Darmstadt, Germany). TLC analysis was performed on Merck 0.25-millimeter Silicagel 60 F_254_ plates. Melting points were determined on a hot stage microscope apparatus (ATM-01, AS ONE, Osaka, Japan) and were uncorrected. NMR spectra were recorded on a JEOLRESONANCE EXC-400 or ECX-500 spectrometer (JEOL, Akishima, Japan) operating at 400 or 500 MHz for ^1^H-NMR and 100 and 125 MHz for ^13^C NMR. Chemical shifts (δ ppm) in CDCl_3_ were reported downfield from TMS (= 0) for ^1^H-NMR. For ^13^C-NMR, chemical shifts were reported in the scale relative to CDCl_3_ (77.00 ppm) as an internal reference. Mass spectra were measured on a JMS-T100LC spectrometer (JEOL, Akishima, Japan). HPLC data were obtained on a SHIMADZU (Kyoto, Japan) HPLC system (consisting of the following: LC-20AT, CMB20A, CTO-20AC and detector SPD-20A measured at 254 nm) using Chiracel AD-H or Ad-3 column (Daicel, Himeji, Japan, 25 cm) at 25 °C. Optical rotations were measured on a JASCO DIP-370 (Na lamp, 589 nm). All NMR spectra figures could be found in Supplementary Materials.

**Methyl (*R*)-5-hydroxy-3-oxotetradecanoate [(*R*)-6]** [25].


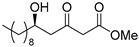


Ti(O*i*Pr)_4_ (0.11 mL, 0.36 mmol) was added to the solution of (*S*)-BINOL (103 mg, 0.36 mmol) and LiCl (31 mg, 0.72 mmol) in THF (18 mL) at 20–25 °C under an Ar atmosphere. After 5 min, decanal (938 mg, 6.00 mmol) was added to the solution, at same temperature. After 10 min, 1,3-bis(TMS)diene **5** (2.97 g, 63% purity, 7.20 mmol) was added to the solution, at same temperature, followed by being stirred for 4 h. The mixture was quenched with 1 M HCl aq. (6 mL), and stirred for 10 min. The mixture was extracted three times with AcOEt, combined organic phase was washed with water, brine, dried (Na_2_SO_4_) and concentrated. The obtained crude oil was purified using SiO_2_-column chromatography (hexane-AcOEt = 10/1) to give the desired product **(*R*)-6** (1.28 g, 78%).

Pale yellow crystal; mp 34–36 °C, [α]_D_^24^ −25.5 (*c* 1.05, CHCl_3_) [lit. [25] [α]_D_^unknown^ −22 (*c* 1, CHCl_3_)]; 98% ee; HPLC analysis (AD-H, flow rate 1.00 mL/min, solvent: hexane/*i*PrOH = 15:1) t_R_(racemic) = 6.79, 7.45, 10.44, and 11.10 min. t_R_[(*R*)-form] = 7.44 and 11.07 min.; ^1^H NMR (500 MHz, CDCl_3_): δ = 0.88 (t, *J* = 6.9 Hz, 3H), 1.22–1.54 (m, 16H), 2.64 (dd, *J* = 9.2 Hz, 17.2 Hz, 1H), 2.73 (dd, *J* = 2.9 Hz, 17.2 Hz, 1H), 3.50 (s, 2H), 3.75 (s, 3H), 4.05–4.10 (m, 1H); ^13^C NMR (125 MHz, CDCl_3_): δ = 14.1, 22.6, 25.4, 29.3, 29.5, 29.5, 29.5, 31.8, 36.5, 49.6 (2C), 52.4, 67.5, 167.3, 203.6.; IR (neat): ν_max_ = 3468, 2926, 2855, 1748, 1715, 1439, 1321, 1238, 1159, 1012, 760.; HRMS (ESI): *m*/*z* calculated for C_15_H_28_O_4_ [*M* + Na]^+^ 295.1885; found: 295.1912.

**Methyl (*S*)-5-hydroxy-3-oxotetradecanoate [(*S*)-6]** [26].


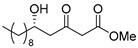


Following a similar procedure for the synthesis of **(*R*)-6**, the reaction using Ti(O*i*Pr)_4_ (0.11 mL, 0.36 mmol), (*R*)-BINOL (103 mg, 0.36 mmol), LiCl (31 mg, 0.72 mmol), decanal (938 mg, 6.00 mmol), 1,3-bis(TMS)diene **5** (2.97 g, 63% purity, 7.20 mmol) and THF (18 mL) gave the desired product **(*S*)-6** (1.16 g, 71%).

Pale yellow crystal; mp 35–36 °C, [α]_D_^25^ +25.7 (*c* 1.07, CHCl_3_) [lit. [26] [α]_D_^25^ +26.2 (*c* 1.0, CHCl_3_)]; 99% ee; HPLC analysis (AD-H, flow rate 1.00 mL/min, solvent: hexane/*i*PrOH = 15:1) t_R_(racemic) = 6.79, 7.45, 10.44 and 11.10 min. t_R_[(*S*)-form] = 6.62 and 10.06 min.

**Methyl (*R*)-5-hydroxy-3-oxopentadecanoate [(*R*)-8]**.


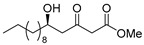


Following a similar procedure for the synthesis of **(*R*)-6**, the reaction using Ti(O*i*Pr)_4_ (0.11 mL, 0.36 mmol), (*S*)-BINOL (103 mg, 0.36 mmol), LiCl (31 mg, 0.72 mmol), undecanal (1.02 g, 6.00 mmol), 1,3-bis(TMS)diene **5** (2.57 g, 73% purity, 7.20 mmol) and THF (18 mL) gave the desired product **(*R*)-8** (1.19 g, 69%).

Pale yellow crystal; mp 45–46 °C, [α]_D_^22^ −26.3 (*c* 1.04, CHCl_3_); 99% ee; HPLC analysis (AD-H, flow rate 1.00 mL/min, solvent: hexane/*i*PrOH = 15:1) t_R_(racemic) = 6.82, 7.53, 10.35 and 10.95 min. t_R_[(*R*)-form] = 7.55 and 10.98 min.; ^1^H NMR (500 MHz, CDCl_3_): δ = 0.88 (t, *J* = 6.9 Hz, 3H), 1.23–1.54 (m, 18H), 2.65 (dd, *J* = 9.2 Hz, 17.2 Hz, 1H), 2.71 (d, *J* = 3.4 Hz, 1H), 2.74 (dd, *J* = 2.9 Hz, 17.2 Hz, 1H), 3.50 (s, 2H), 3.75 (s, 3H), 4.05–4.10 (m, 1H); ^13^C NMR (125 MHz, CDCl_3_): δ = 14.1, 22.6, 25.4, 29.3, 29.5, 29.6(3C), 31.9, 36.4, 49.6(2C), 52.4, 67.5, 167.4, 203.7.; IR (neat): ν_max_ = 3374, 2953, 2849, 1734, 1715, 1470, 1331, 1150, 1059, 1034, 910.; HRMS (ESI): *m*/*z* calculated for C_16_H_30_O_4_ [*M* + Na]^+^ 309.2042; found: 309.2062.

**Methyl (*S*)-5-hydroxy-3-oxopentadecanoate [(*S*)-8]**.


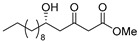


Following a similar procedure for the synthesis of **(*R*)-6**, the reaction using Ti(O*i*Pr)_4_ (0.11 mL, 0.36 mmol), (*R*)-BINOL (103 mg, 0.36 mmol), LiCl (31 mg, 0.72 mmol), undecanal (1.02 g, 6.00 mmol), 1,3-bis(TMS)diene **5** (2.57 g, 73% purity, 7.20 mmol) and THF (18 mL) gave the desired product **(*S*)-8** (1.16 g, 67%).

Pale yellow crystal; mp 44–45 °C, [α]_D_^22^ +25.2 (*c* 1.08, CHCl_3_); 99% ee; HPLC analysis (AD-H, flow rate 1.00 mL/min, solvent: hexane/*i*PrOH = 15:1) t_R_(racemic) = 6.82, 7.53, 10.35 and 10.95 min. t_R_[(*S*)-form] = 6.31 and 9.56 min.

**Methyl (*R*)-5-hydroxy-3-oxohexadecanoate [(*R*)-9]**.


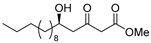


Following a similar procedure for the synthesis of **(*R*)-6**, the reaction using Ti(OiPr)_4_ (0.11 mL, 0.36 mmol), (*S*)-BINOL (103 mg, 0.36 mmol), LiCl (31 mg, 0.72 mmol), dodecanal (1.11 g, 6.00 mmol), 1,3-bis (TMS)diene **5** (2.57 g, 73% purity, 7.20 mmol) and THF (18 mL) gave the desired product **(*R*)-9** (1.19 g, 66%).

Pale yellow crystal mp 45–46 °C, [α]_D_^22^ −24.1 (*c* 1.04, CHCl_3_); 99% ee; HPLC analysis (AD-H, flow rate 1.00 mL/min, solvent: hexane/*i*PrOH = 15:1) t_R_(racemic) = 5.90, 6.56, 8.86 and 9.45 min. t_R_[(*R*)-form] = 6.58 and 9.47 min.; ^1^H NMR (500 MHz, CDCl_3_): δ = 0.88 (t, *J* = 6.9 Hz, 3H), 1.21–1.54 (m, 20H), 2.65 (dd, *J* = 9.2 Hz, 17.8 Hz, 1H), 2.74 (dd, *J* = 2.9 Hz, 17.8 Hz, 1H), 3.50 (s, 2H), 3.75 (s, 3H), 4.05–4.10 (m, 1H); ^13^C NMR (125 MHz, CDCl_3_): δ = 14.1, 22.7, 25.4, 29.3, 29.5, 29.6(2C), 29.6, 29.6, 31.9, 36.4, 49.6(2C), 52.4, 67.5, 167.3, 203.7.; IR (neat): ν_max_ = 3347, 2914, 2849, 1736, 1717, 1470, 1437, 1335, 1209, 1138, 854.; HRMS (ESI): *m*/*z* calculated for C_17_H_32_O_4_ [*M* + Na]^+^ 323.2198; found: 323.2211.

**Methyl (*S*)-5-hydroxy-3-oxohexadecanoate [(*S*)-9]** [14].


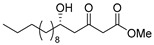


Following a similar procedure for the synthesis of **(*R*)-6**, the reaction using Ti(OiPr)_4_ (0.11 mL, 0.36 mmol), (*R*)-BINOL (103 mg, 0.36 mmol), LiCl (31 mg, 0.72 mmol), dodecanal (1.11 g, 6.00 mmol), 1,3-bis(TMS)diene **5** (2.57 g, 73% purity, 7.20 mmol) and THF (18 mL) gave the desired product **(*S*)-9** (1.27 g, 70%).

Pale yellow crystal; mp 46–47 °C, [α]_D_^24^ +24.7 (*c* 1.02, CHCl_3_) [lit. [14] [α]_D_^unknown^ −26.2 (*c* 1.9, CHCl_3_)]; 99% ee; HPLC analysis (AD-H, flow rate 1.00 mL/min, solvent: hexane/*i*PrOH = 15:1) t_R_(racemic) = 5.90, 6.56, 8.86 and 9.45 min. t_R_[(*S*)-form] = 5.93 and 8.88 min.

**Methyl (*R*)-5-hydroxy-3-oxopentadec-14-enoate [(*R*)-10]**.


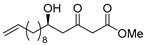


Following a similar procedure for the synthesis of **(*R*)-6**, the reaction using Ti(O*i*Pr)_4_ (0.11 mL, 0.36 mmol), (*S*)-BINOL (103 mg, 0.36 mmol), LiCl (31 mg, 0.72 mmol), 10-undecenal (1.01 g, 6.00 mmol), 1,3-bis(TMS)diene **5** (2.97 g, 63% purity, 7.20 mmol) and THF (18 mL) gave the desired product **(*R*)-10** (1.39 g, 81%).

Pale yellow crystal; mp 29–30 °C, [α]_D_^24^ −24.5 (*c* 1.02, CHCl_3_); >99% ee; HPLC analysis (AD-H, flow rate 1.00 mL/min, solvent: hexane/*i*PrOH = 15:1) t_R_(racemic) = 7.33, 8.10, 11.39 and 12.18 min. t_R_[(*R*)-form] = 8.01 and 12.00 min.; ^1^H NMR (500 MHz, CDCl_3_): δ = 1.22–1.54 (m, 14H), 2.04 (q, *J* = 6.9 Hz, 2H), 2.65 (dd, *J* = 9.2 Hz, 17.8 Hz, 1H), 2.69 (brs, 1H), 2.74 (dd, *J* = 2.9 Hz, 17.8 Hz, 1H), 3.50 (s, 2H), 3.75 (s, 3H), 4.56–4.10 (m, 1H), 4.93 (dd, *J* = 1.2 Hz, 10.3 Hz, 1H), 5.00 (dd, *J* = 1.7 Hz, 17.2 Hz, 1H), 5.77–5.85 (m, 1H); ^13^C NMR (125 MHz, CDCl_3_): δ = 25.4, 28.8, 29.0, 29.3, 29.4, 29.4, 33.7, 36.4, 49.6 (2C), 52.4, 67.5, 114.1, 139.2, 167.3, 203.6.; IR (neat): ν_max_ = 3503, 2926, 2855, 1748, 1715, 1639, 1437, 1406, 1325, 1269, 910. HRMS (ESI): *m*/*z* calculated for C_16_H_28_O_4_ [*M* + Na]^+^ 307.1885; found: 307.1872.

**Methyl (*S*)-5-hydroxy-3-oxopentadec-14-enoate [(*S*)-10]**.


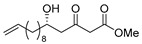


Following a similar procedure for the synthesis of **(*R*)-6**, the reaction using Ti(O*i*Pr)_4_ (0.11 mL, 0.36 mmol), (*R*)-BINOL (103 mg, 0.36 mmol), LiCl (31 mg, 0.72 mmol), 10-undecenal (1.01 g, 6.00 mmol), 1,3-bis(TMS)diene **5** (2.97 g, 63% purity, 7.20 mmol) and THF (18 mL) gave the desired product **(*S*)-9** (1.38 g, 81%).

Pale yellow crystal; mp 30–31 °C, [α]_D_^24^ +24.9 (*c* 1.13, CHCl_3_); >99% ee; HPLC analysis (AD-H, flow rate 1.00 mL/min, solvent: hexane/*i*PrOH = 15:1) t_R_(racemic) = 7.33, 8.10, 11.39 and 12.18 min. t_R_[(*S*)-form] = 7.32 and 11.37 min.

**(*R*)-6-Nonyldihydro-2*H*-pyran-2,4(3*H*)-dione [(*R*)-7]** [32].


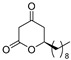


(*R*)-Aldol adduct **(*R*)-6** (640 mg, 2.35 mmol) was added to a stirred 1M KOH aq. solution (2.4 mL) at 20–25 °C under an Ar atmosphere and the mixture was stirred at same temperature for 3 h. The resulting mixture was quenched with 1M HCl aq. solution, which was extracted twice with AcOEt. The combined organic phase was washed with water, brine, dried (Na_2_SO_4_) and concentrated. The obtained crude crystal **(*R*)-7** (526 mg, 93%) was used for the next step without purification.

Pale yellow crystal; mp 82–83 °C, ^1^H NMR (500 MHz, CDCl_3_): δ = 0.87 (t, *J* = 6.9 Hz, 3H), 1.16–1.57 (m, 14H), 1.64–1.71 (m, 1H), 1.78–1.85 (m, 1H), 2.46 (dd, *J* = 11.5 Hz, 18.3 Hz, 1H)), 2.69 (dd, *J* = 2.3 Hz, 18.3 Hz, 1H), 3.42 (d, *J* = 18.9 Hz, 1H), 3.55 (d, *J* = 18.9 Hz, 1H), 4.58–4.64 (m, 1H); ^13^C NMR (125 MHz, CDCl_3_): δ =14.1, 22.6, 24.7, 29.2, 29.2, 29.4, 29.4, 31.8, 34.6, 43.5, 47.0, 75.6, 167.3, 200.1.; IR (neat): ν_max_ =2922, 2855, 2666, 1697, 1585, 1389, 1285, 1248, 1043, 833. HRMS (ESI): *m*/*z* calculated for C_14_H_24_O_3_ [*M* + Na]^+^ 263.1623; found: 263.1604.

**(*S*)-6-Nonyldihydro-2*H*-pyran-2,4(3*H*)-dione [(*S*)-7]** [32].


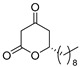


Following a similar procedure for the synthesis of **(*R*)-7**, the reaction using (*S*)-aldol adduct **(*S*)-8** (817 mg, 3.00 mmol) gave the desired product **(*S*)-7** (710 mg, 98%).

Pale yellow crystal; mp 82–83 °C.

**(*R*)-6-Decyldihydro-2*H*-pyran-2,4(3*H*)-dione [(*R*)-11]**.


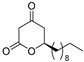


Following a similar procedure for the synthesis of **(*R*)-7**, the reaction using aldol adduct **(*R*)-10** (859 mg, 3.00 mmol) gave the desired product **(*R*)-11** (733 mg, 96%).

Pale yellow crystal; mp 72–74 °C; ^1^H NMR (500 MHz, CDCl_3_): δ = 0.88 (t, *J* = 6.9 Hz, 3H), 1.27–1.57 (m, 16H), 1.66–1.73 (m, 1H), 1.80–1.87 (m, 1H), 2.48 (dd, *J* = 11.5 Hz, 18.3 Hz, 1H), 2.71 (dd, *J* = 2.3 Hz, 18.3 Hz, 1H), 3.44 (d, *J* = 18.9 Hz, 1H), 3.57 (d, *J* = 18.9 Hz, 1H), 4.60–4.66 (m, 1H); ^13^C NMR (125 MHz, CDCl_3_): δ = 14.1, 22.7, 24.7, 29.2, 29.3, 29.4, 29.5, 29.5, 31.9, 34.5, 43.5, 47.0, 75.5, 167.3, 200.2.; IR (neat): ν_max_ = 2922, 2853, 1697, 1587, 1315, 1285, 1244, 910, 831, 735.; HRMS (ESI): *m*/*z* calculated for C_15_H_26_O_3_ [*M* + Na]^+^ 277.1780; found: 277.1770.

**(*S*)-6-Decyldihydro-2*H*-pyran-2,4(3*H*)-dione [(*S*)-11]**.


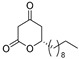


Following a similar procedure for the synthesis of **(*R*)-7**, the reaction using (*S*)-aldol adduct **(*S*)-8** (859 mg, 3.00 mmol) gave the desired product **(*S*)-11** (745 mg, 98%).

Pale yellow crystal; mp 73–74 °C.

**(*R*)-6-Undecyldihydro-2*H*-pyran-2,4(3*H*)-dione [(*R*)-12]**.


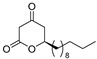


Following a similar procedure for the synthesis of **(*R*)-7**, the reaction using aldol adduct **(*R*)-9** (901 mg, 3.00 mmol) and 1M KOH aq. solution (3.0 mL) gave the desired product **(*S*)-12** (748 mg, 93%).

Pale yellow crystal; mp 80–81 °C, ^1^H NMR (500 MHz, CDCl_3_): δ = 0.88 (t, *J* = 6.9 Hz, 3H), 1.21–1.54 (m, 18H), 1.66–1.73 (m, 1H), 1.80–1.87 (m, 1H), 2.47 (dd, *J* = 11.5 Hz, 18.3 Hz, 1H), 2.71 (dd, *J* = 2.9 Hz, 18.3 Hz, 1H), 3.44 (d, *J* = 18.9 Hz, 1H), 3.57 (d, *J* = 18.9 Hz, 1H), 4.60–4.65 (m, 1H); ^13^C NMR (125 MHz, CDCl_3_): δ = 14.1, 22.7, 24.7, 29.2, 29.3, 29.4, 29.5, 29.6(2C), 31.9, 34.5, 43.5, 47.0, 75.5, 167.3, 200.2.; IR (neat): ν_max_ = 2920, 2853, 1697, 1585, 1470, 1389, 1285, 1043, 907, 831, 737.; HRMS (ESI): *m*/*z* calculated for C_16_H_28_O_3_ [*M* + Na]^+^ 291.1936; found: 291.1949.

**(*S*)-6-Undecyldihydro-2*H*-pyran-2,4(3*H*)-dione [(*S*)-12]**.


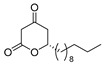


Following a similar procedure for the synthesis of **(*R*)-7**, the reaction using aldol adduct **(*S*)-9** (901 mg, 3.00 mmol) and 1M KOH aq. solution (3.0 mL) gave the desired product **(*S*)-12** (733 mg, 91%).

Pale yellow crystal; mp 77–79 °C.

**(*R*)-6-(Dec-9-en-1-yl)dihydro-2*H*-pyran-2,4(3*H*)-dione [(*R*)-13]**.


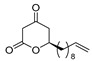


Following a similar procedure for the synthesis of **(*R*)-7**, the reaction using (*R*)-aldol adduct **(*R*)-10** (853 mg, 3.00 mmol) gave the desired product **(*R*)-13** (709 mg, 94%).

Pale yellow crystal; mp 69–71 °C; ^1^H NMR (500 MHz, CDCl_3_): δ = 1.22–1.59 (m, 12H), 1.66–1.73 (m, 1H), 1.80–1.87 (m, 1H), 2.02–2.07 (m, 2H), 2.47 (dd, *J* = 11.5 Hz, 18.3 Hz, 1H), 2.71 (dd, *J* = 2.9 Hz, 18.3 Hz, 1H), 3.44 (d, *J* = 18.9 Hz, 1H), 3.57 (d, *J* = 18.9 Hz, 1H), 4.60–4.65 (m, 1H), 4.94 (dquin *J* = 1.2 Hz, 10.3 Hz, 1H), 5.00 (ddd *J* = 1.7 Hz, 4.0 Hz, 17.2 Hz, 1H), 5.77–5.85 (m, 1H); ^13^C NMR (125 MHz, CDCl_3_): δ = 24.7, 28.8, 29.0, 29.2, 29.3 (2C), 33.7, 34.5, 43.5, 47.0, 75.5, 114.2, 139.1, 167.3, 200.2.; IR (neat): ν_max_ = 2924, 2855, 1697, 1585, 1389, 1285, 1244, 908, 833, 737. HRMS (ESI): *m*/*z* calculated for C_15_H_24_O_3_ [*M* + Na]^+^ 275.1623; found: 275.1638.

**(*S*)-6-(Dec-9-en-1-yl)dihydro-2*H*-pyran-2,4(3*H*)-dione [(*S*)-13]**.


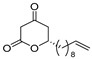


Following a similar procedure for the synthesis of **(*R*)-7**, the reaction using (*S*)-aldol adduct **(*S*)-10** (853 mg, 3.00 mmol) and 1M KOH aq. solution (3.0 mL) gave the desired product **(*S*)-13** (732 mg, 97%).

Pale yellow crystal; mp 68–70 °C.

**(*R*)-3-Butyryl-4-hydroxy-6-nonyl-5,6-dihydro-2*H*-pyran-2-one [(*R*)-*Plymuthipyranone* B: (*R*)-4b]** [16].


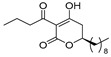


1-Ethyl-3-(3-dimethylaminopropyl)carbodiimide hydrochloride (EDCI•HCl) (115 mg, 0.60 mmol) was added to a stirred suspension of pyrone **(*R*)-7** (120 mg, 0.50 mmol), butanoic acid (0.055 mL, 0.60 mmol) and DMAP (73 mg, 0.60 mmol) in CH_2_Cl_2_ (1.5 mL) at 0–5 °C under an Ar atmosphere, and the mixture was stirred for 15 h at 20–25 °C. The resulting mixture was quenched with 1 M-HCl aq. solution, which was extracted twice with AcOEt. The combined organic phase was washed with water, brine, dried (Na_2_SO_4_) and concentrated. The obtained crude oil was purified using SiO_2_-column chromatography (hexane/AcOEt = 20:1) to give the desired product [(*R*)-*Plymuthipyranone* B: **(*R*)-4b**; 95% ee, 103 mg, 66%].

Pale yellow crystal; mp 45–46 °C, [α]_D_^27^ −23.9 (*c* 0.73, MeOH) [lit. [16] [α]_D_^unknown^ −22 (*c* 0.041, MeOH)]; 95% ee; HPLC analysis (OJ-H, flow rate 1.00 mL/min, solvent: hexane/*i*PrOH = 50:1) t_R_(racemic) = 7.89 and 11.14 min. t_R_[(*R*)-form] = 7.82 min.; ^1^H NMR (500 MHz, CDCl_3_): δ = 0.88 (t, *J* = 6.9 Hz, 3H), 1.00 (t, *J* = 7.5 Hz, 3H), 1.22–1.53 (m, 14H), 1.61–1.84 (m, 4H), 2.60 (dd, *J* = 4.0 Hz, 17.2 Hz, 1H), 2.67 (dd, *J* = 11.5 Hz, 17.2 Hz, 1H), 2.94–3.09 (m, 2H), 4.33–4.38 (m, 1H), 17.90 (s, 1H); ^13^C NMR (125 MHz, CDCl_3_): δ = 13.9, 14.2, 18.5, 22.7, 24.8, 29.3 (2C), 29.5, 29.5, 31.9, 34.7, 38.0, 40.4, 73.9, 103.3 164.4, 195.2, 204.4.; IR (neat): ν_max_ = 2957, 2926, 2855, 1717, 1558, 1466, 1275, 1070, 910, 735.

**(*S*)-3-Butyryl-4-hydroxy-6-nonyl-5,6-dihydro-2*H*-pyran-2-one [(*S*)-*Plymuthipyranone* B: (*S*)-4b]** [16].


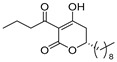


Following a similar procedure for the synthesis of (*R*)-*Plymuthipyranone* B [**(*R*)-4b**], the reaction using EDCI•HCl (115 mg, 0.80 mmol), pyrone **(*S*)-7** (160 mg, 0.67 mmol), butanoic acid (0.073 mL, 0.80 mmol), DMAP (98 mg, 0.80 mmol) gave the desired product (*S*)-*Plymuthipyranone* B **[(*S*)-4b]** (149 mg, 72%).

Pale yellow crystal; mp 43–44 °C, [α]_D_^27^ +25.7 (*c* 0.67, MeOH) [lit. [16] [α]_D_^unknown^ +33 (*c* 2.4, CH_2_Cl_2_)]; >97% ee; HPLC analysis (OJ-H, flow rate 1.00 mL/min, solvent: hexane/*i*PrOH = 50:1) t_R_(racemic) = 7.89 and 11.14 min. t_R_[(*S*)-form] = 10.99 min.

**(*R*)-3-Butyryl-6-decyl-4-hydroxy-5,6-dihydro-2*H*-pyran-2-one [(*R*)-4c]**.


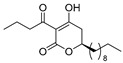


Following a similar procedure for the synthesis of **(*R*)-4b**, the reaction using EDCI•HCl (230 mg, 1.20 mmol), pyrone **(*R*)-11** (254 mg, 1.00 mmol), butyric acid (0.11 mL, 1.20 mmol), DMAP (147 mg, 1.20 mmol) and CH_2_Cl_2_ (3.0 mL) gave the desired product **(*R*)-4c** (236 mg, 73%).

Pale yellow crystal; mp 47–48 °C, [α]_D_^24^ –25.2 (*c* 1.03, MeOH); 97% ee; HPLC analysis (OJ-H, flow rate 1.00 mL/min, solvent: hexane/*i*PrOH = 50:1) t_R_(racemic) = 7.54 and 10.66 min. t_R_[(*R*)-form] = 7.45 min.; ^1^H NMR (500 MHz, CDCl_3_): δ = 0.88 (t, *J* = 6.9 Hz, 3H), 1.00 (t, *J* = 7.5 Hz, 3H), 1.21–1.54 (m, 16H), 1.61–1.84 (m, 4H), 2.57–2.70 (m, 2H), 2.94–3.09 (m, 2H), 4.33–4.39 (m, 1H), 17.93 (s, 1H); ^13^C NMR (125 MHz, CDCl_3_): δ = 13.8, 14.1, 18.3, 22.7, 24.6, 29.3, 29.3, 29.4, 29.5, 29.5, 31.9, 34.6, 37.8, 40.3, 73.8, 103.2, 164.4, 195.1, 204.4. IR (neat): ν_max_ = 2963, 2920, 2849, 1709, 1692, 1557, 1466, 1281, 1081, 912, 762; HRMS (ESI): *m*/*z* calculated for C_19_H_32_O_4_ [*M* + Na]^+^ 347.2198; found: 347.2181.

***(S*)-3-Butyryl-6-decyl-4-hydroxy-5,6-dihydro-2*H*-pyran-2-one [(*S*)-4c]**.


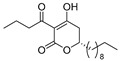


Following a similar procedure for the synthesis of **(*R*)-4b**, the reaction using EDCI•HCl (230 mg, 1.20 mmol), pyrone **(*S*)-11** (254 mg, 1.00 mmol), butyric acid (0.11 mL, 1.20 mmol), DMAP (147 mg, 1.20 mmol) and CH_2_Cl_2_ (3.0 mL) gave the desired product **(*S*)-4c** (246 mg, 76%).

Pale yellow crystal; mp 47–48 °C, [α]_D_^21^ +24.9 (*c* 1.03, MeOH); 99% ee; HPLC analysis (OJ-H, flow rate 1.00 mL/min, solvent: hexane/*i*PrOH = 50:1) t_R_(racemic) = 7.54 and 10.66 min. t_R_[(*S*)-form] = 10.47 min.

**(*R*)-3-Butyryl-6-undecyl-4-hydroxy-5,6-dihydro-2*H*-pyran-2-one [(*R*)-4d]**.


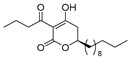


Following a similar procedure for the synthesis of (*R*)-*plymuthipyranone* B [**(*R*)-4b**], the reaction using EDCI•HCl (230 mg, 1.20 mmol), pyrone **(*R*)-12** (268 mg, 1.00 mmol), butanoic acid (0.11 mL, 1.20 mmol), DMAP (147 mg, 1.20 mmol) and CH_2_Cl_2_ (3.0 mL) gave the desired product **(*R*)-4d** (230 mg, 68%).

Pale yellow crystal; mp 50–51 °C, [α]_D_^24^ –23.5 (*c* 1.00, MeOH); 97% ee; HPLC analysis (OJ-H, flow rate 1.00 mL/min, solvent: hexane/*i*PrOH = 50:1) t_R_(racemic) = 7.09 and 9.90 min. t_R_[(*R*)-form] = 6.98 min.; ^1^H NMR (500 MHz, CDCl_3_): δ = 0.88 (t, *J* = 6.9 Hz, 3H), 1.00 (t, *J* = 7.5 Hz, 3H), 1.23–1.54 (m, 18H), 1.61–1.83 (m, 4H), 2.57–2.70 (m, 2H), 2.94–3.09 (m, 2H), 4.33–4.39 (m, 1H), 17.92 (s, 1H); ^13^C NMR (125 MHz, CDCl_3_): δ = 13.8, 14.1, 18.4, 22.7, 24.7, 29.3, 29.3, 29.4, 29.5, 29.6(2C), 31.9, 34.6, 37.9, 40.3, 73.9, 103.2, 164.4, 195.1, 204.4 IR (neat): ν_max_ = 2920, 2849, 1707, 1688, 1558, 1468, 1070, 912, 735.; HRMS (ESI): *m*/*z* calculated for C_20_H_34_O_4_ [*M* + Na]^+^ 361.2355; found: 361.2337.

***(S*)-3-Butyryl-6-undecyl-4-hydroxy-5,6-dihydro-2*H*-pyran-2-one [(*S*)-4d]**.


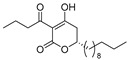


Following a similar procedure for the synthesis of (*R*)-*plymuthipyranone* B [**(*R*)-4b**], the reaction using EDCI•HCl (230 mg, 1.20 mmol), pyrone **(*S*)-12** (268 mg, 1.00 mmol), butanoic acid (0.11 mL, 1.20 mmol), DMAP (147 mg, 1.20 mmol) and CH_2_Cl_2_ (3.0 mL) gave the desired product **(*S*)-4d** (222 mg, 66%). 

Pale yellow crystal; mp 48–49 °C, [α]_D_^26^ +23.5 (*c* 0.93, MeOH); 98% ee; HPLC analysis (OJ-H, flow rate 1.00 mL/min, solvent: hexane/*i*PrOH = 50:1) t_R_(racemic) = 7.09 and 9.90 min. t_R_[(*S*)-form] = 9.74 min.

**(*R*)-3-Butyryl-6-(dec-9-en-1-yl)-4-hydroxy-5,6-dihydro-2*H*-pyran-2-one [(*R*)-4e]**.


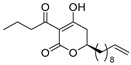


Following a similar procedure for the synthesis of (*R*)-*plymuthipyranone* B [**(*R*)-4b**], the reaction using EDCI•HCl (230 mg, 1.20 mmol), pyrone **(*R*)-13** (252 mg, 1.00 mmol), butanoic acid (0.11 mL, 1.20 mmol) and DMAP (147 mg, 1.20 mmol) gave the desired product **(*R*)-4e** (237 mg, 73%).

Pale yellow crystal; mp 34–35 °C, [α]_D_^25^ -25.1 (*c* 0.64, MeOH); 97% ee; HPLC analysis (OJ-H, flow rate 1.00 mL/min, solvent: hexane/*i*PrOH = 50:1), t_R_(racemic) = 10.06 and 15.51 min. t_R_[(*R*)-form] = 9.96 min.; ^1^H NMR (500 MHz, CDCl_3_): δ = 1.00 (t, *J* = 7.5 Hz, 3H), 1.22–1.56 (m, 12H), 1.61–1.83 (m, 4H), 2.02–2.06 (m, 2H), 2.60 (dd, *J* = 3.4 Hz, 17.2 Hz, 1H), 2.68 (dd, *J* = 11.5 Hz, 17.2 Hz, 1H), 2.94–3.09 (m, 2H), 4.33–4.39 (m, 1H), 4.92–5.02 (m, 2H), 5.77–5.85 (m, 1H), 17.92 (s, 1H); ^13^C NMR (125 MHz, CDCl_3_): δ = 13.8, 18.4, 24.6, 28.9, 29.0, 29.2, 29.3, 29.3, 33.8, 34.6, 37.9, 40.3, 73.8, 103.2, 114.1, 139.1, 164.3, 195.1, 204.4.; IR (neat): ν_max_ = 2926, 2857, 1715, 1558, 1464, 1273, 1069, 910, 767; HRMS (DART): *m*/*z* calculated for C_19_H_30_O_4_ [*M* + H]^+^ 323.2222; found: 323.2205.

**(*S*)-3-Butyryl-6-(dec-9-en-1-yl)-4-hydroxy-5,6-dihydro-2*H*-pyran-2-one [(*S*)-4e]**.


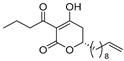


Following a similar procedure for the synthesis of (*R*)-*Plymuthipyranone* B, the reaction using EDCI•HCl (230 mg, 1.20 mmol), pyrone **(*S*)-13** (252 mg, 1.00 mmol), butanoic acid (0.11 mL, 1.20 mmol) and DMAP (147 mg, 1.20 mmol) gave the desired product **(*S*)-4e** (227 mg, 70%).

Pale yellow crystal; mp 34–35 °C, [α]_D_^26^ +24.9 (*c* 0.75, MeOH); 98% ee; HPLC analysis (OJ-H, flow rate 1.00 mL/min, solvent: hexane/*i*PrOH = 50:1), t_R_(racemic) = 10.06 and 15.51 min. t_R_[(*S*)-form] = 15.39 min.

### 3.2. Bioassay

ATCC 43300 and ATCC 33591 were purchased from the American Type Culture Collection and were stored at −80 °C in a freezer by using the Microbank system (IWAKI & CO., Ltd., Tokyo, Japan). For each experiment, a bacterial strain was cultured overnight on mannitol salt agar plates to confirm the purity and viability of the microbe.

The minimal inhibitory concentration (MIC) of synthetics were determined by microdilution method using Mueller–Hinton broth according to Clinical and Laboratory Standards Institute (CLSI) guidelines [33,34]. Briefly, after diluting the suspension of bacteria equivalent to 1 × 10^6^ colony-forming units (CFU)/mL with Mueller–Hinton II broth (Becton, Dickinson and Company, MD, USA) with 17.5 mg/L of calcium, the dilution (50 μL) was applied into 96-well plate, which included synthetics or vancomycin (Cayman Chemical Company, MI, USA) (50 μL) at concentrations of 0.5–256 μg/mL. Final inoculum concentration was approximately 5 × 10^5^ CFU/mL. Synthetics concentration was 0.25–128 μg/mL. After incubation at 37 °C for 16–20 h, MICs were determined.

## 4. Conclusions

We performed asymmetric total syntheses of all of the both enantiomers of the *anti*-MRSA active plymuthipyranone B and three unnatural analogues. The present synthetic method utilized a privileged asymmetric Mukaiyama aldol addition using Ti(O*i*Pr)_4_/(*S*)- or (*R*)-BINOL catalysis as the key step, originally developed by Soriente and Scettri’s group. The total syntheses were each implemented in three steps and an overall yield of 42–56% with a highly enantiomeric excess (95–99%). The bioassay of the *anti*-MRSA activity against ATCC 43300 and 33591 revealed that natural (*R*)-plymuthipyranone B and (*R*)-undecyl synthetic analogue at the C6 position exhibited the highest activity with low MIC values.

These findings provide new insight into the SAR with the chiral discrimination regarding *anti*-MRSA compounds comprising the 3-acyl-5,6-dihydro-2*H*-pyran-2-one structure.

## Figures and Tables

**Table 1:** Susceptibility testing all four pairs against ATCC 43300 and ATCC 33591.

	MIC (µg/mL)		MIC (µg/mL)		
	ATCC 43300	ATCC 33591	ATCC 43300		ATCC 33591
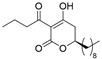 plymuthipyranone B (*R*)-**4b**	4	8	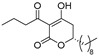 plymuthipyranone B (*S*)-**4b**	16	16
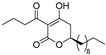 (*R*)-**4c**	4	8	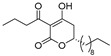 (*S*)-**4c**	2	4
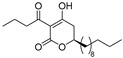 (*R*)-**4d**	2	4	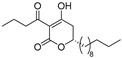 (*S*)-**4d**	4	4
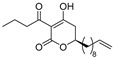 (*R*)-**4e**	16	16	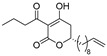 (*S*)-**4e**	16	16
vancomycin	1	1			

## Data Availability

Data is contained within article and supplementary material.

## Supplementary Materials

The following are available online at https://www.mdpi.com/article/10.3390/ph14090938/s1, ^1^H NMR, ^13^C NMR spectra for compounds **6–13, 4b–4e** (Figures S1–S28). **Figure S1.** ^1^H NMR (500 MHz, CDCl_3_) Spectrum of the Compound **6**. **Figure S2.** ^13^C NMR (125 MHz, CDCl_3_) Spectrum of the Compound **6**. **Figure S3.** ^1^H NMR (500 MHz, CDCl_3_) Spectrum of the Compound **8**. **Figure S4.** ^13^C NMR (125 MHz, CDCl_3_) Spectrum of the Compound **8**. **Figure S5.** ^1^H NMR (500 MHz, CDCl_3_) Spectrum of the Compound **9**. **Figure S6.** ^13^C NMR (125 MHz, CDCl_3_) Spectrum of the Compound **9**. **Figure S7.** ^1^H NMR (500 MHz, CDCl_3_) Spectrum of the Compound **10**. **Figure S8.** ^13^C NMR (125 MHz, CDCl_3_) Spectrum of the Compound **10**. **Figure S9.** ^1^H NMR (500 MHz, CDCl_3_) Spectrum of the Compound **7**. **Figure S10.** ^13^C NMR (125 MHz, CDCl_3_) Spectrum of the Compound **7**. **Figure S11.** ^1^H NMR (500 MHz, CDCl_3_) Spectrum of the Compound **11**. **Figure S12.** ^13^C NMR (125 MHz, CDCl_3_) Spectrum of the Compound **11**. **Figure S13.** ^1^H NMR (500 MHz, CDCl_3_) Spectrum of the Compound **12**. **Figure S14.** ^13^C NMR (125 MHz, CDCl_3_) Spectrum of the Compound **12**. **Figure S15.** ^1^H NMR (500 MHz, CDCl_3_) Spectrum of the Compound **13**. **Figure S16.** ^13^C NMR (125 MHz, CDCl_3_) Spectrum of the Compound **13**. **Figure S17.** ^1^H NMR (500 MHz, CDCl_3_) Spectrum of the Compound **4b**. **Figure S18.** ^1^H NMR (500 MHz, CDCl_3_) Spectrum of the Compound **4b**. **Figure S19.** ^13^C NMR (125 MHz, CDCl_3_) Spectrum of the Compound **4b**. **Figure S20.** ^1^H NMR (500 MHz, CDCl_3_) Spectrum of the Compound **4c**. **Figure S21.** ^1^H NMR (500 MHz, CDCl_3_) Spectrum of the Compound **4c**. **Figure S22.** ^13^C NMR (125 MHz, CDCl_3_) Spectrum of the Compound **4c**. **Figure S23.** ^1^H NMR (500 MHz, CDCl_3_) Spectrum of the Compound **4d**. **Figure S24.** ^1^H NMR (500 MHz, CDCl_3_) Spectrum of the Compound **4d**. **Figure S25.** ^13^C NMR (125 MHz, CDCl_3_) Spectrum of the Compound **4d**. **Figure S26.** ^1^H NMR (500 MHz, CDCl_3_) Spectrum of the Compound **4e**. **Figure S27.** ^1^H NMR (500 MHz, CDCl_3_) Spectrum of the Compound **4e**. **Figure S28.** ^13^C NMR (125 MHz, CDCl_3_) Spectrum of the Compound **4e**. Charts of HPLC analyses for compounds **6** (racemic), **(*R*)-6, (*S*)-6, 8** (racemic), **(*R*)-8, (*S*)-8, 9** (racemic), **(*R*)-9, (*S*)-9, 10** (racemic), **(*R*)-10, (*S*)-10, 4b** (racemic), **(*R*)-4b, (*S*)-4b, 4c** (racemic), **(*R*)-4c, (*S*)-4c, 4d** (racemic), **(*R*)-4d, (*S*)-4d, 4e** (racemic), **(*R*)-4e** and **(*S*)-4e**.
