# Supplementary material for: Asymmetric Total Syntheses of Both Enantiomers of Plymuthipyranone B and Its Unnatural Analogues: Evaluation of anti-MRSA Activity and Its Chiral Discrimination
Source: Pharmaceuticals (Basel). 2021 Sep 19;14(9):938. doi: 10.3390/ph14090938 (PMC8465287; doi:10.3390/ph14090938)
Supplement: Supplementary file 1 [file pharmaceuticals-14-00938-s001.zip › pharmaceuticals-1345069-supplementary.pdf]

pharmaceuticals

MDPI

Supporting Information

Asymmetric total syntheses of both enantiomers of plymuthipyranone B and its unnatural analogues: evaluation of *anti*-MRSA activity and its chiral discrimination

Mizuki Moriyama[1], Xiaoxi Liu[2], Yuki Enoki[2], Kazuaki Matsumoto[2] and Yoo Tanabe[1,*]

[1] Department of Chemistry, School of Science and Technology, Kwansei Gakuin University, 2-1 Gakuen, Sanda, Hyogo 669-1337, Japan; ixu18409@kwansei.ac.jp (M.M.)
[2] Division of Pharmacodynamics, Keio University Faculty of Pharmacy, 1-5-30 Shibakoen, Minato-ku, Tokyo 105-8512, Japan; aganeiliu@gmail.com (X.L.); enoki-yk@pha.keio.ac.jp (Y.E.); matsumoto-kz@pha.keio.ac.jp (K.M.)
* Correspondence: tanabe@kwansei.ac.jp; Tel. +81-795-565-8394

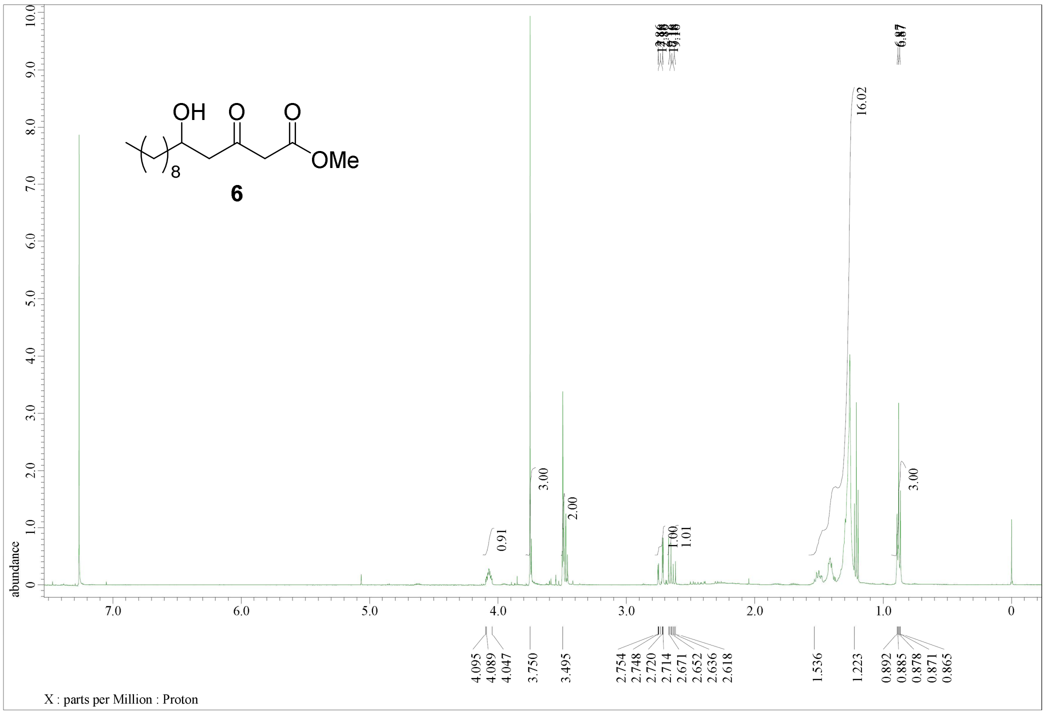

Figure S1. ¹H NMR (500 MHz, $CDCl_3$) Spectrum of the Compound 6.

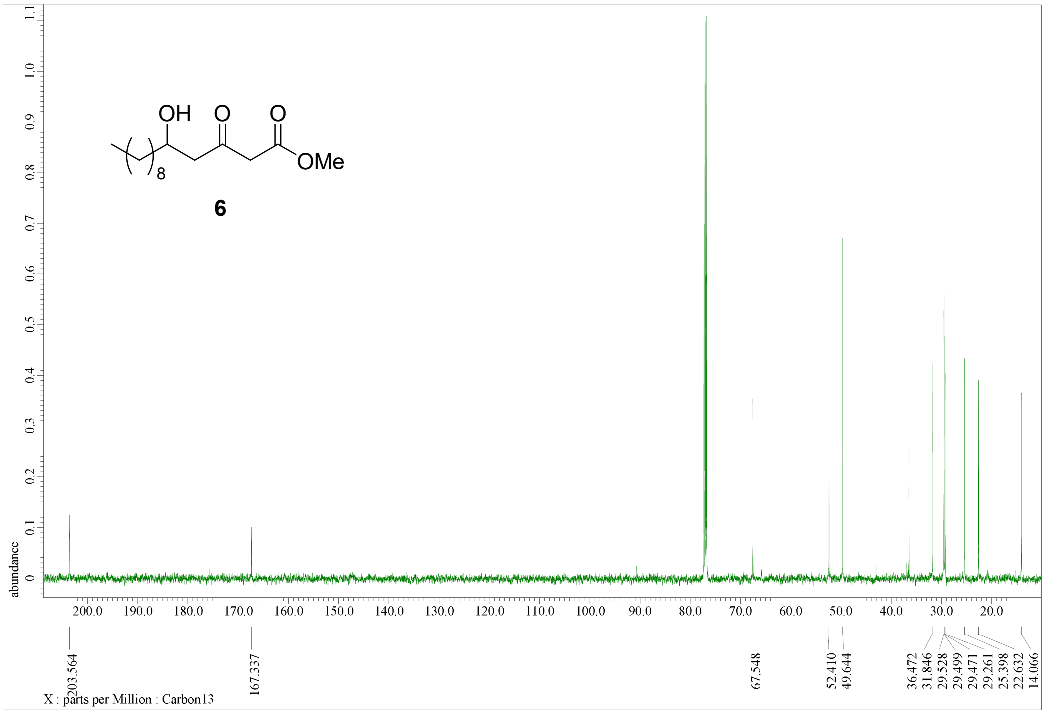

Figure S2. ¹³C NMR (125 MHz, $CDCl_3$) Spectrum of the Compound 6.

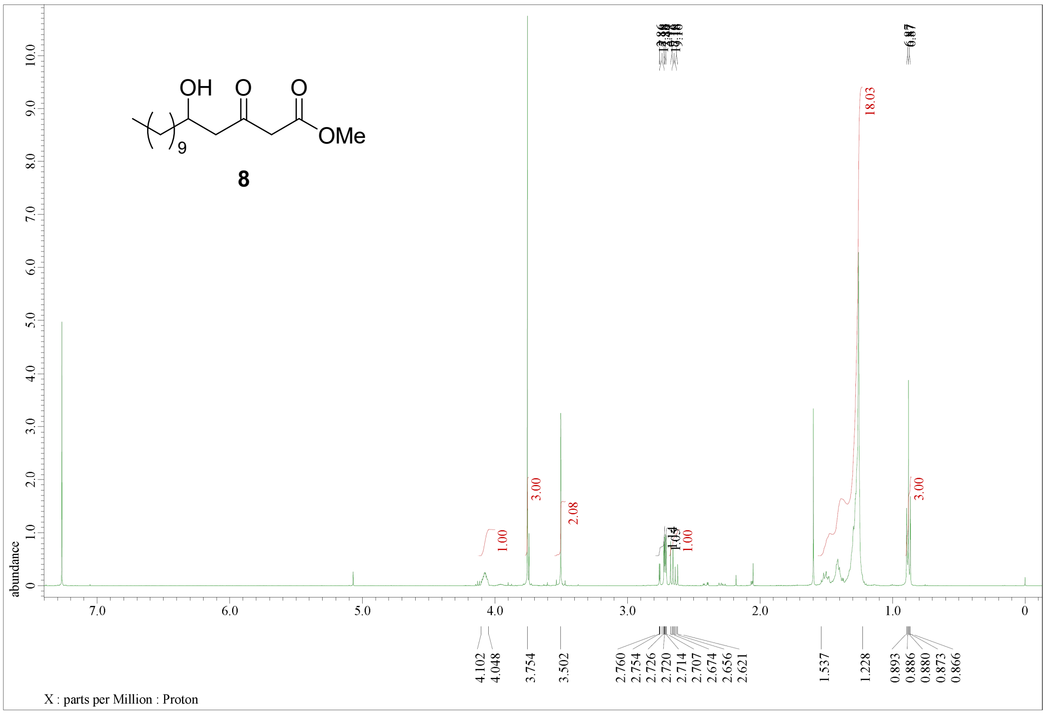

Figure S3. ^{1}H NMR (500 MHz, $CDCl_3$) Spectrum of the Compound 8.

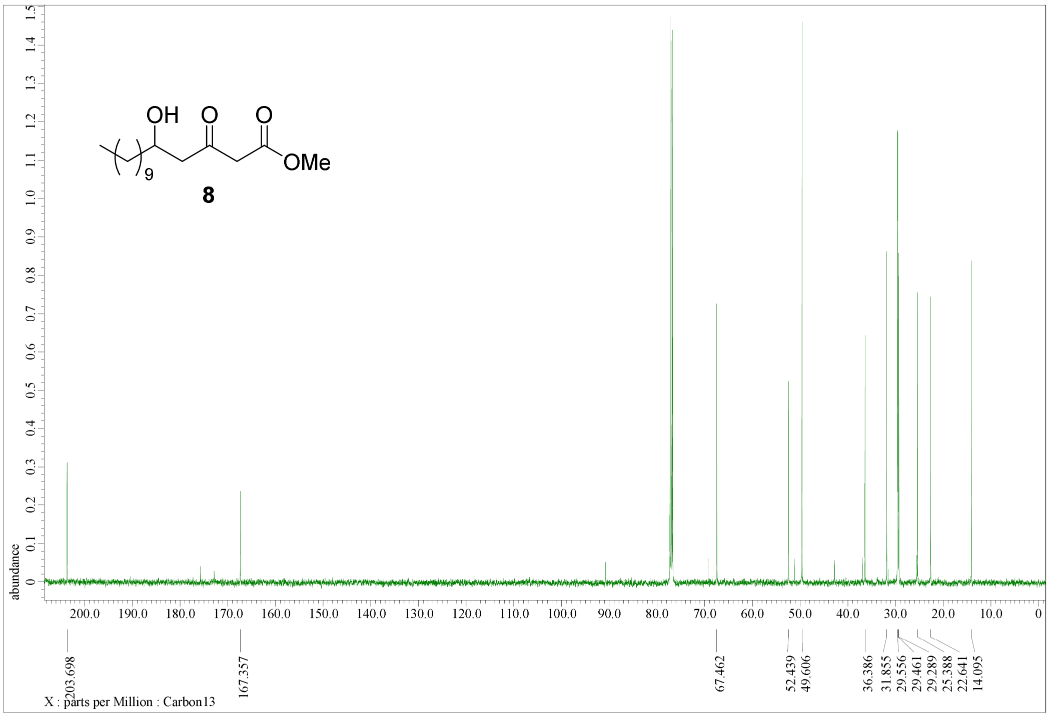

Figure S4. ^{13}C NMR (125 MHz, $CDCl_3$) Spectrum of the Compound 8.

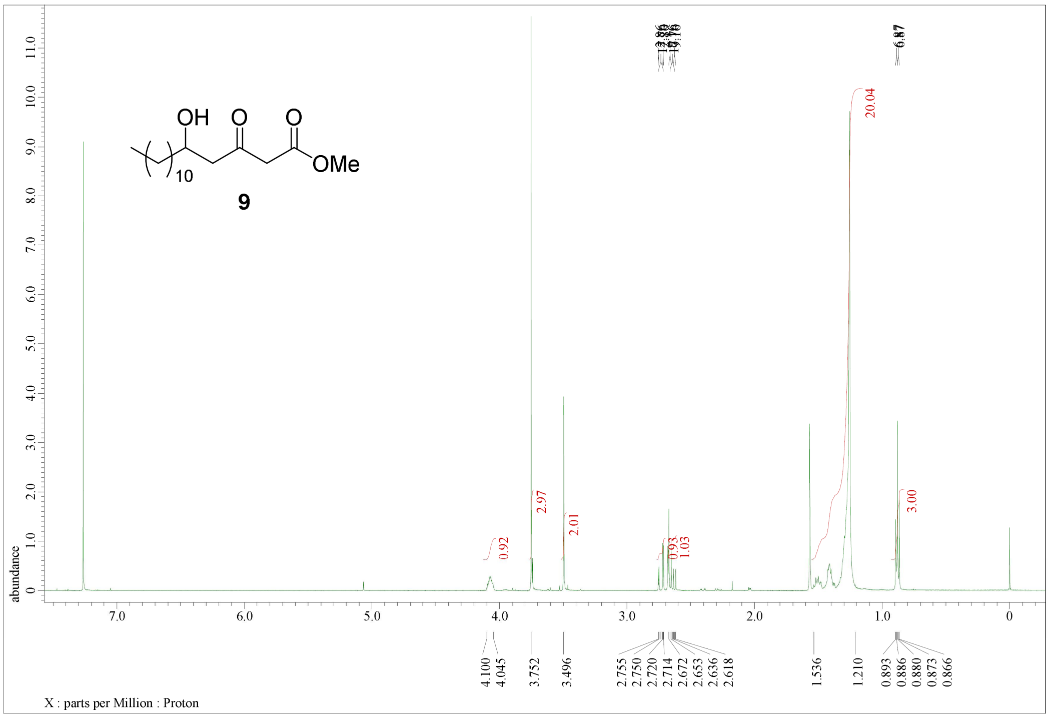

Figure S5. ^{1}H NMR (500 MHz, $CDCl_3$) Spectrum of the Compound 9.

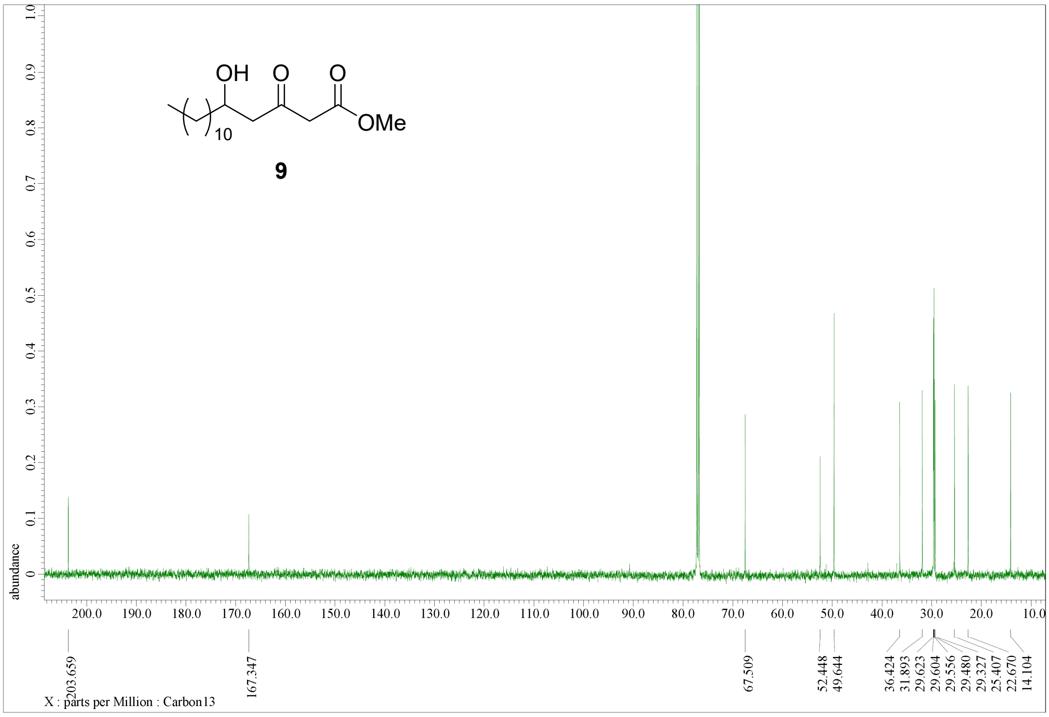

Figure S6. ^{13}C NMR (125 MHz, $CDCl_3$) Spectrum of the Compound 9.

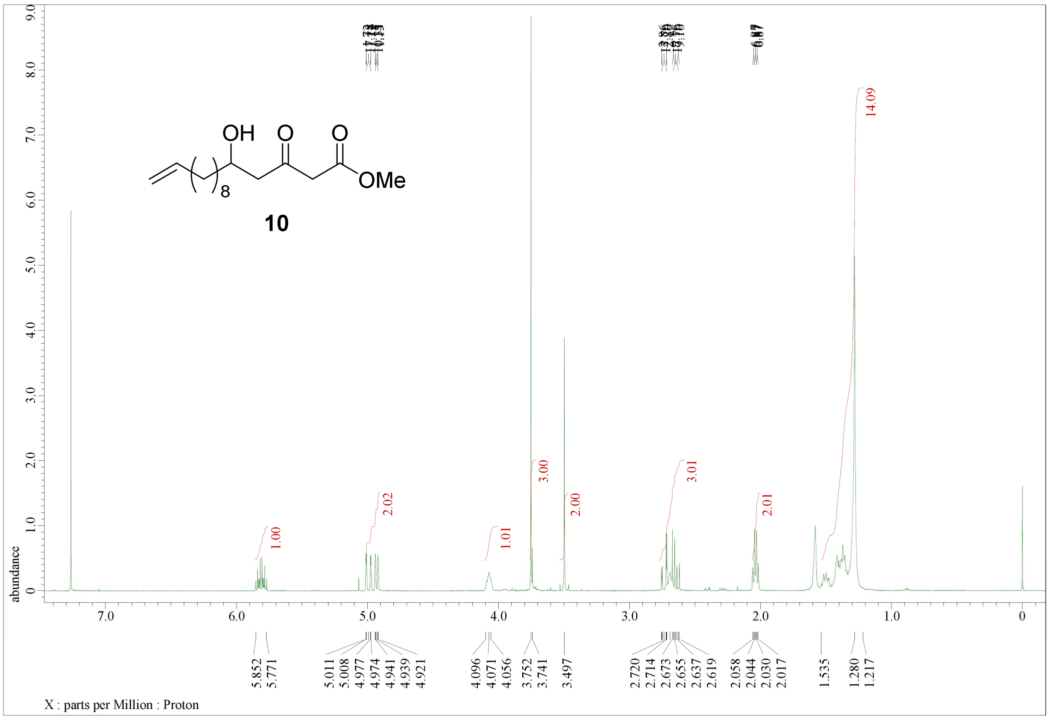

Figure S7. ^{1}H NMR (500 MHz, $CDCl_3$) Spectrum of the Compound 10.

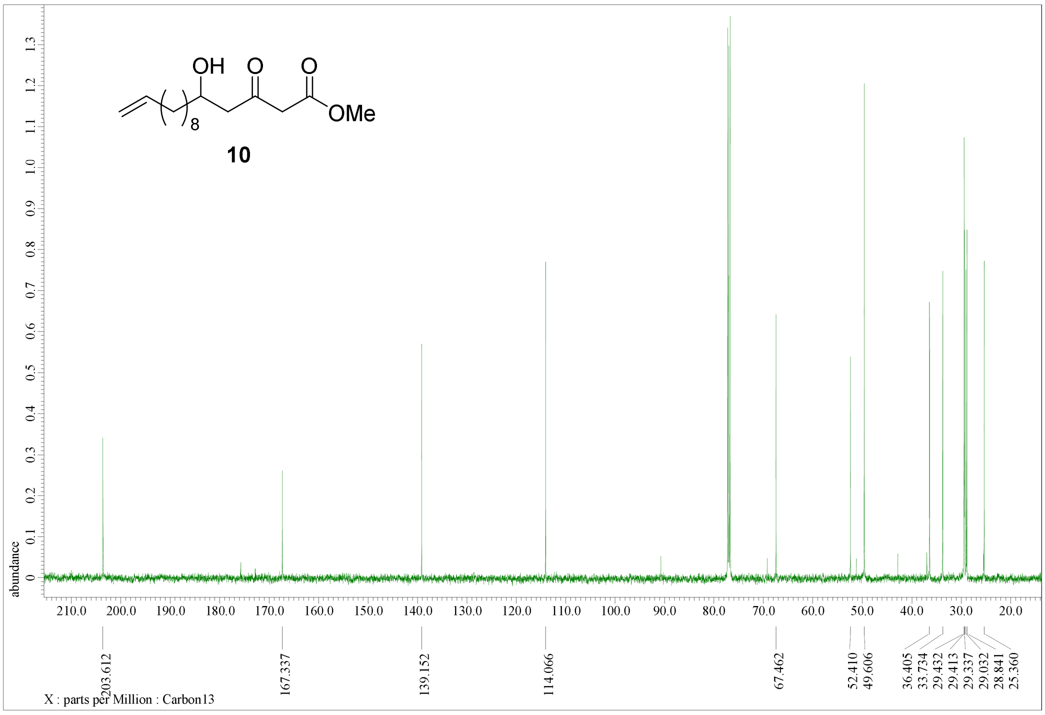

Figure S8. ^{13}C NMR (125 MHz, $CDCl_3$) Spectrum of the Compound 10.

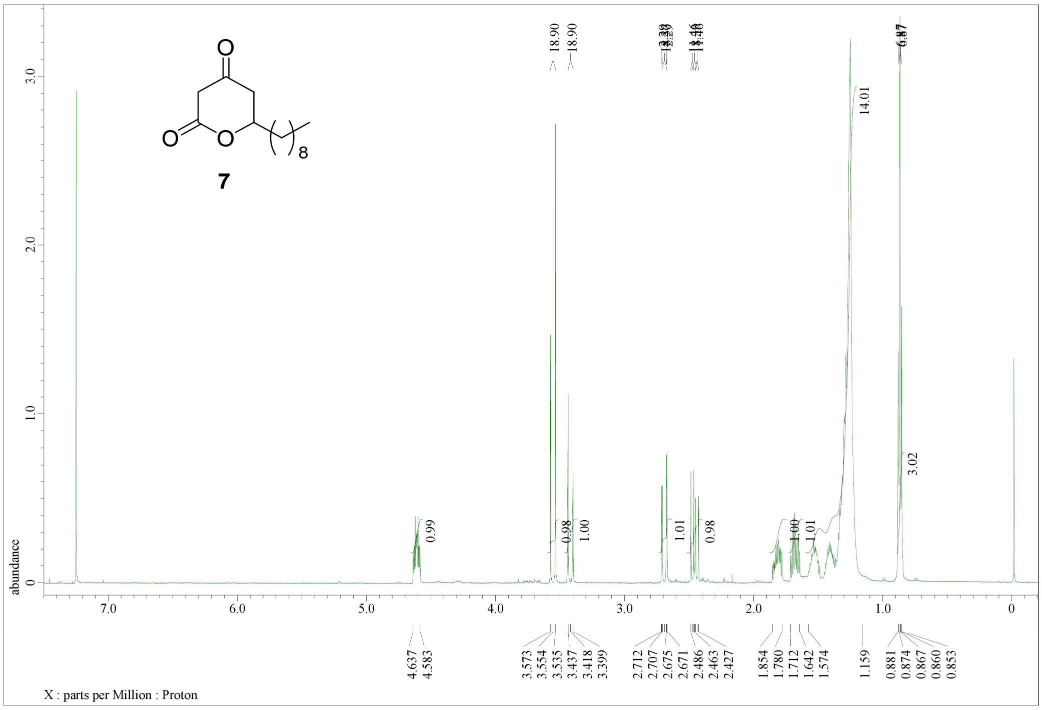

Figure S9. ^1H NMR (500 MHz, $CDCl_3$) Spectrum of the Compound 7.

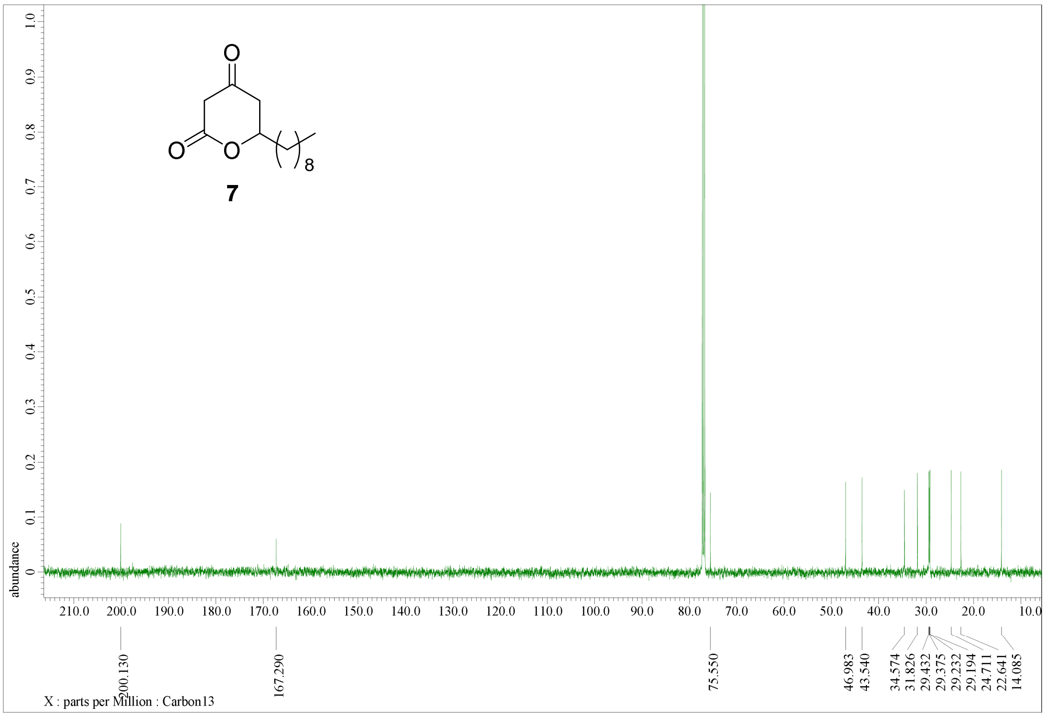

Figure S10. ^{13}C NMR (125 MHz, $CDCl_3$) Spectrum of the Compound 7.

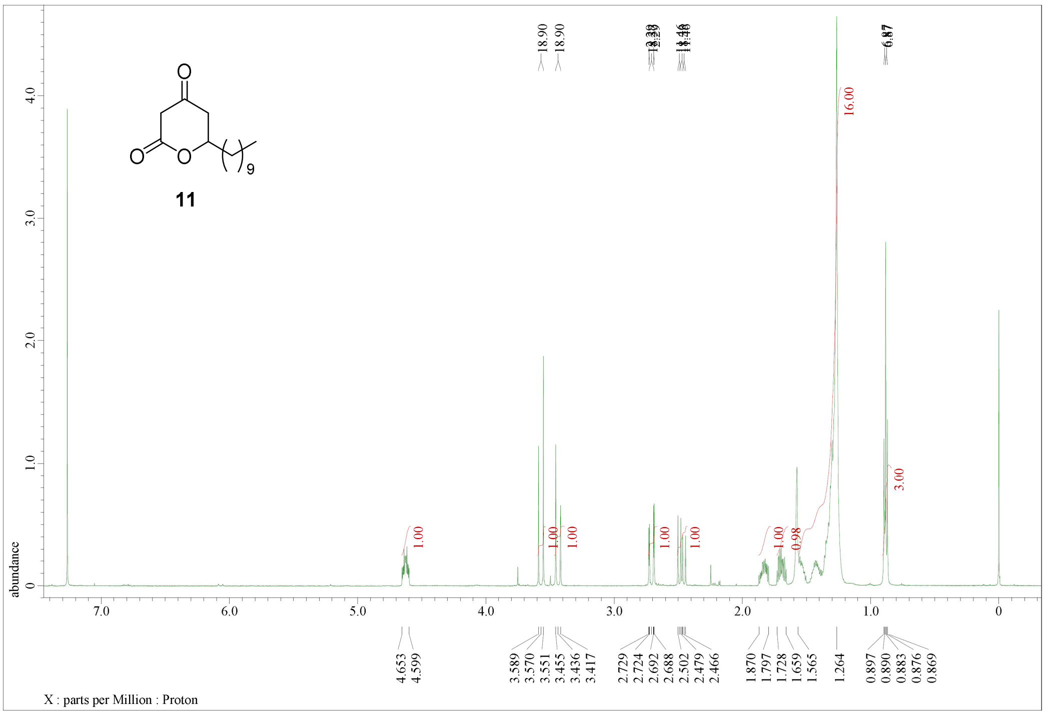

Figure S11. ^{1}H NMR (500 MHz, $CDCl_3$) Spectrum of the Compound 11.

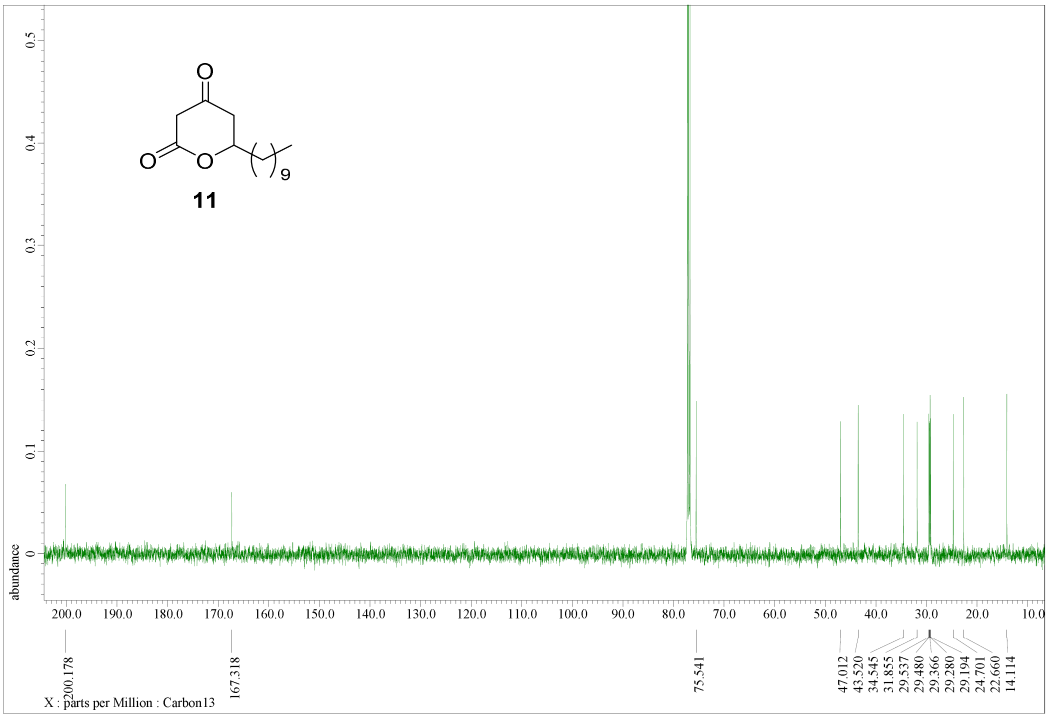

Figure S12. ^{13}C NMR (125 MHz, $CDCl_3$) Spectrum of the Compound 11.

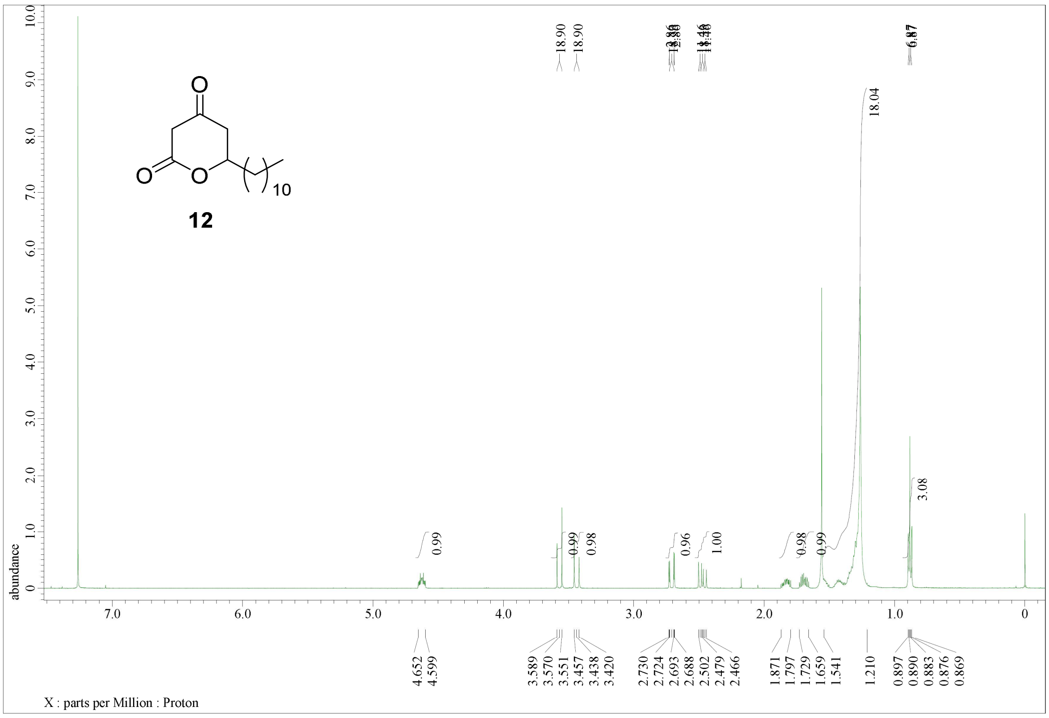

Figure S13. ^{1}H NMR (500 MHz, $CDCl_3$) Spectrum of the Compound 12.

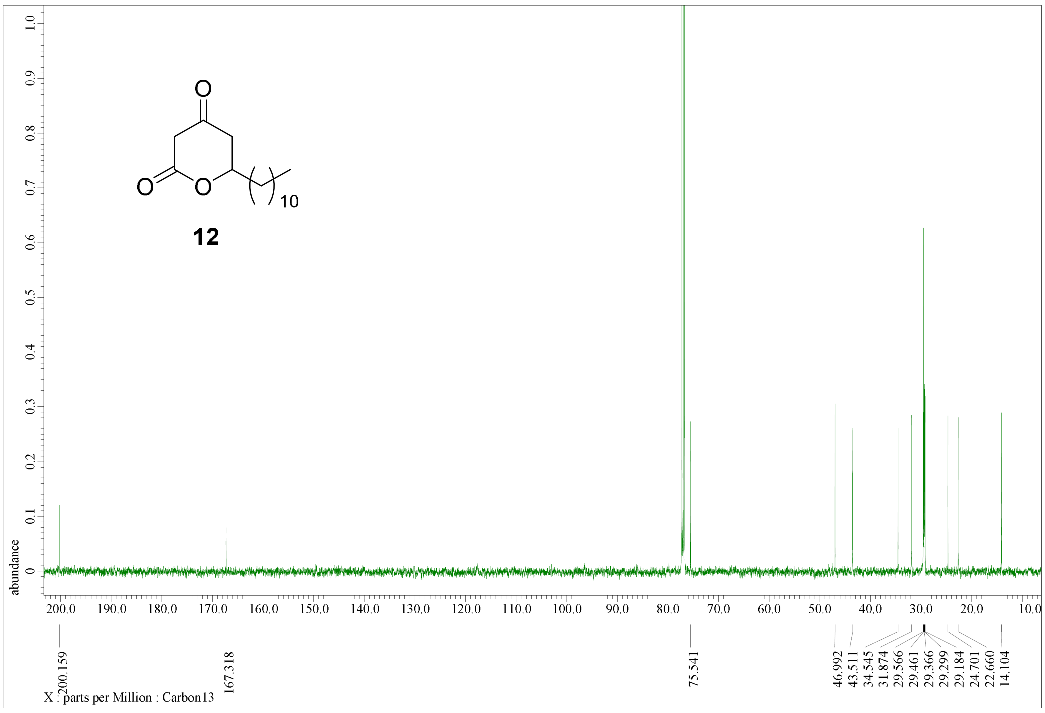

Figure S14. ^{13}C NMR (125 MHz, $CDCl_3$) Spectrum of the Compound 12.

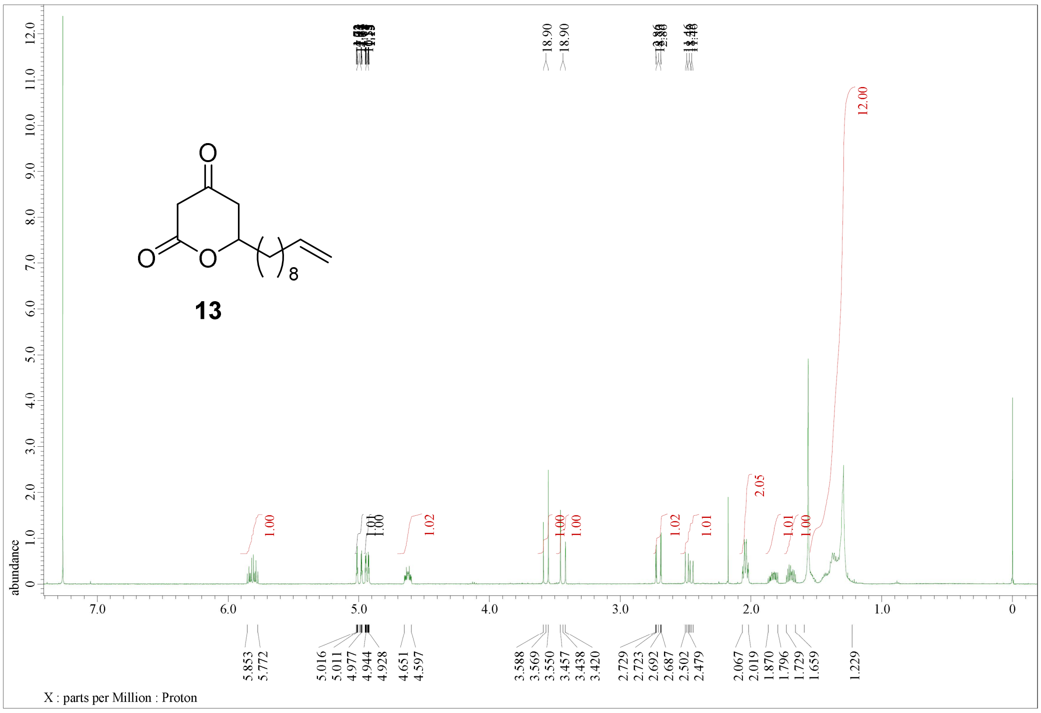

Figure S15. ^{1}H NMR (500 MHz, $CDCl_3$) Spectrum of the Compound 13.

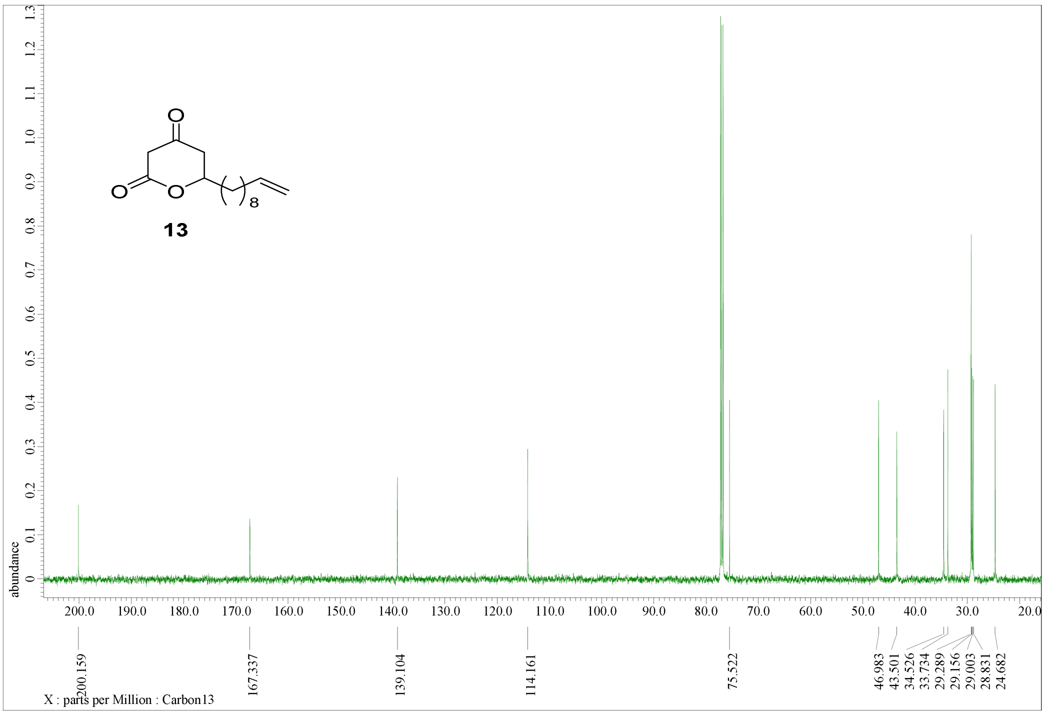

Figure S16. ^{13}C NMR (125 MHz, $CDCl_3$) Spectrum of the Compound 13.

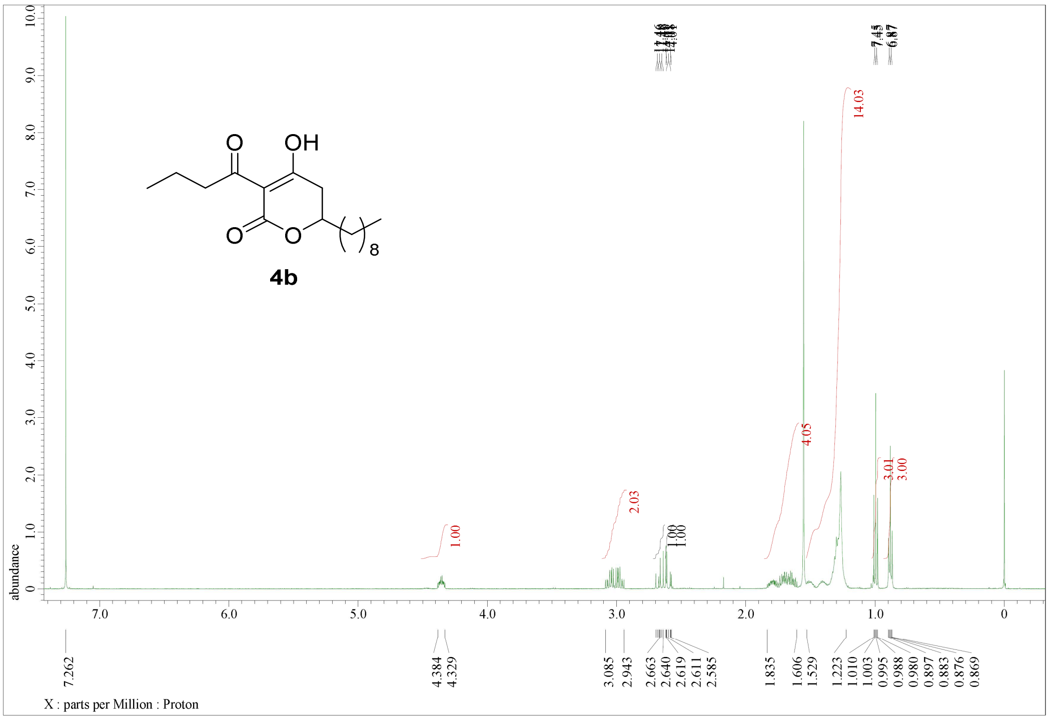

Figure S17. ^{1}H NMR (500 MHz, $CDCl_3$) Spectrum of the Compound 4b.

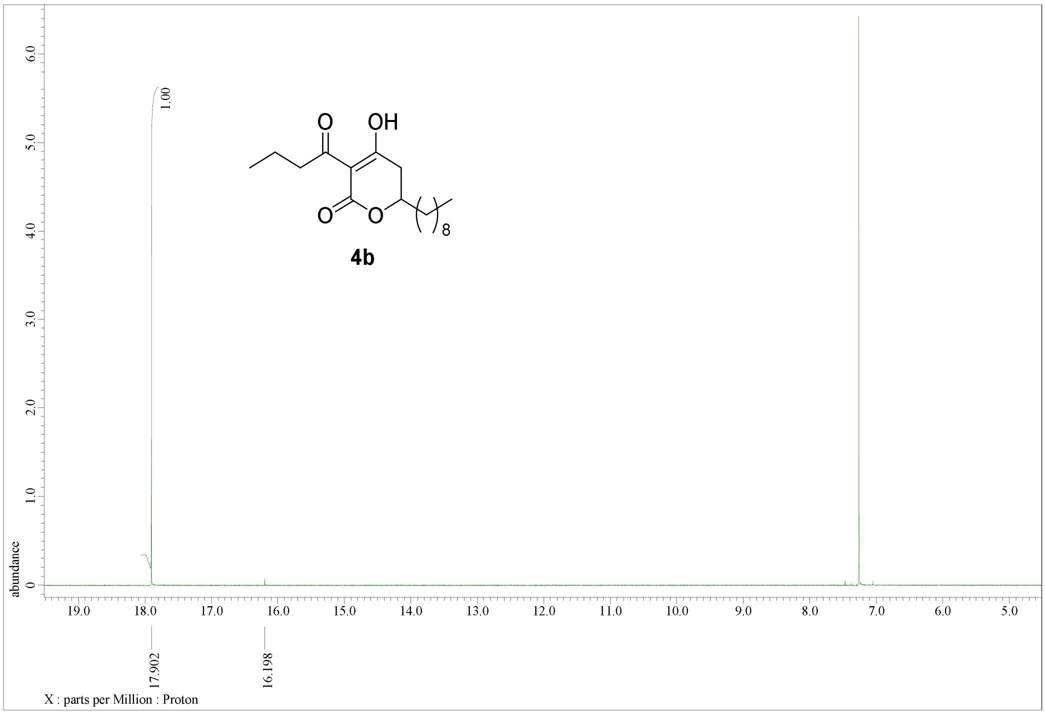

Figure S18. ^{1}H NMR (500 MHz, $CDCl_3$) Spectrum of the Compound 4b.

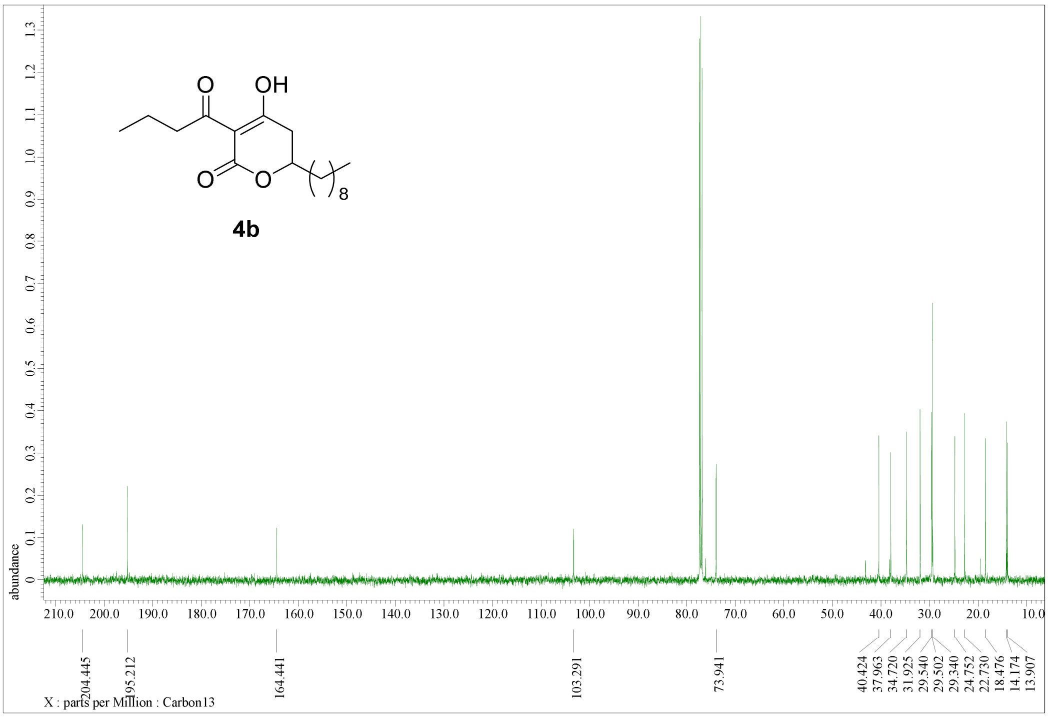

Figure S19. ^{13}C NMR (125 MHz, $CDCl_3$) Spectrum of the Compound 4b.

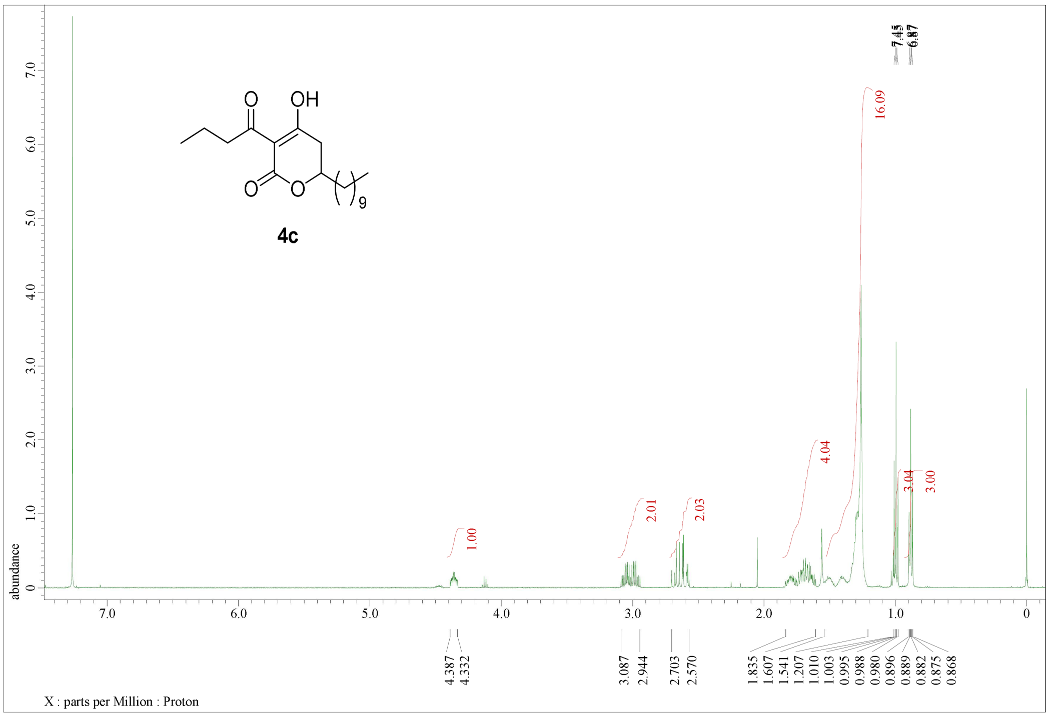

Figure S20. ^{1}H NMR (500 MHz, $CDCl_3$) Spectrum of the Compound 4c.

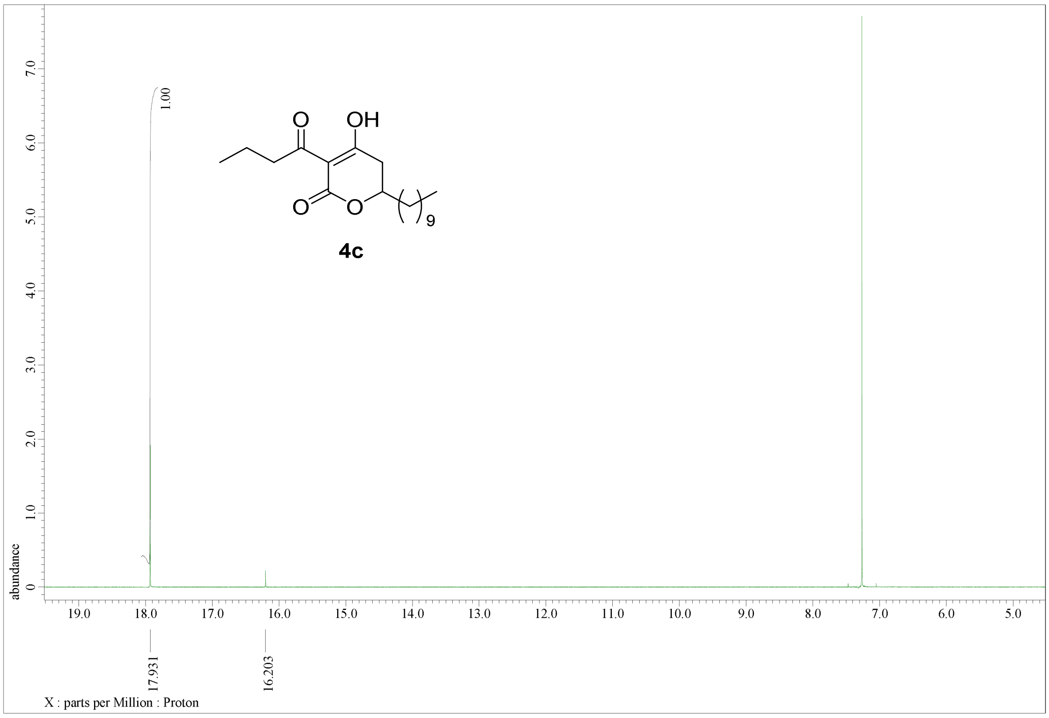

Figure S21. ^{1}H NMR (500 MHz, $CDCl_3$) Spectrum of the Compound 4c.

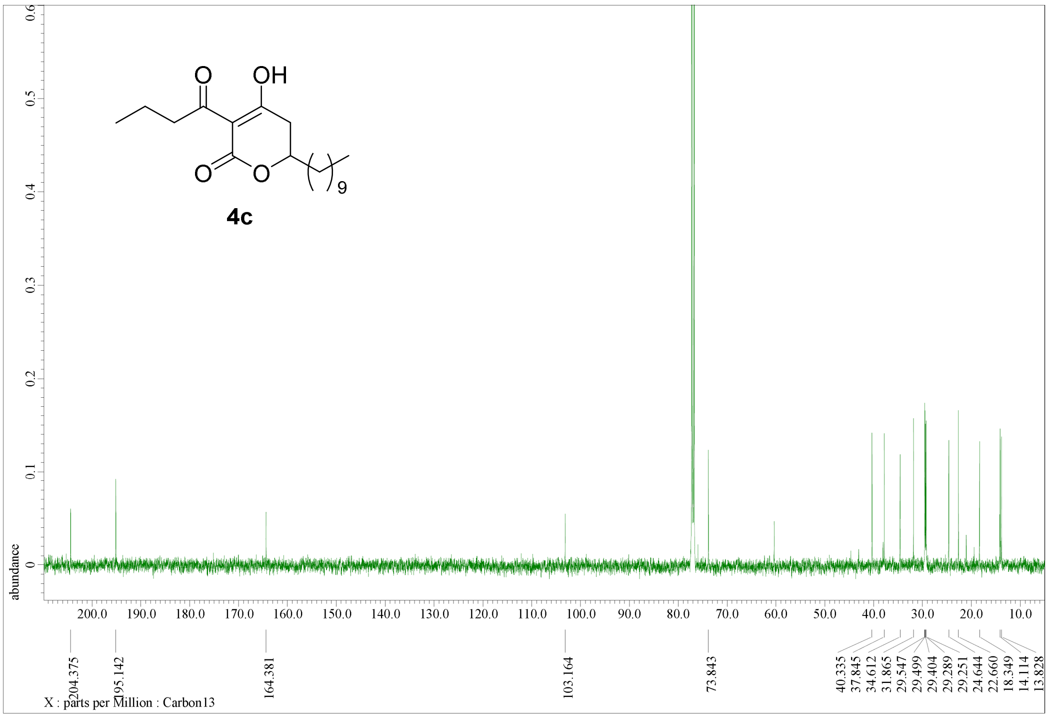

Figure S22. ^{13}C NMR (125 MHz, $CDCl_3$) Spectrum of the Compound 4c.

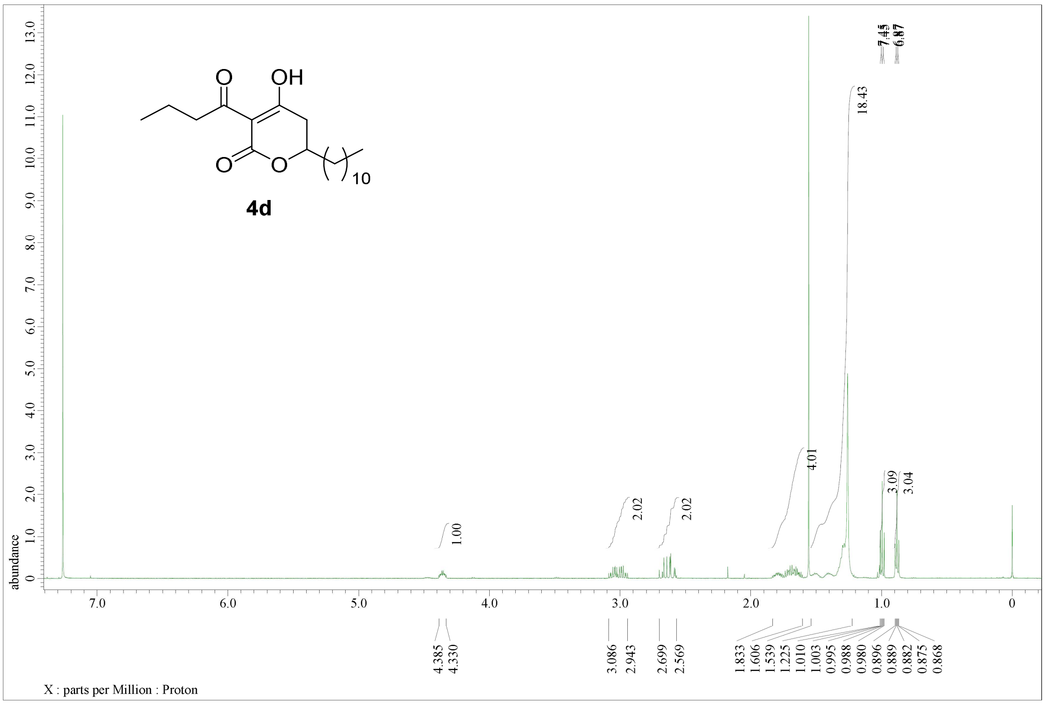

Figure S23. ^{1}H NMR (500 MHz, $CDCl_3$) Spectrum of the Compound 4d.

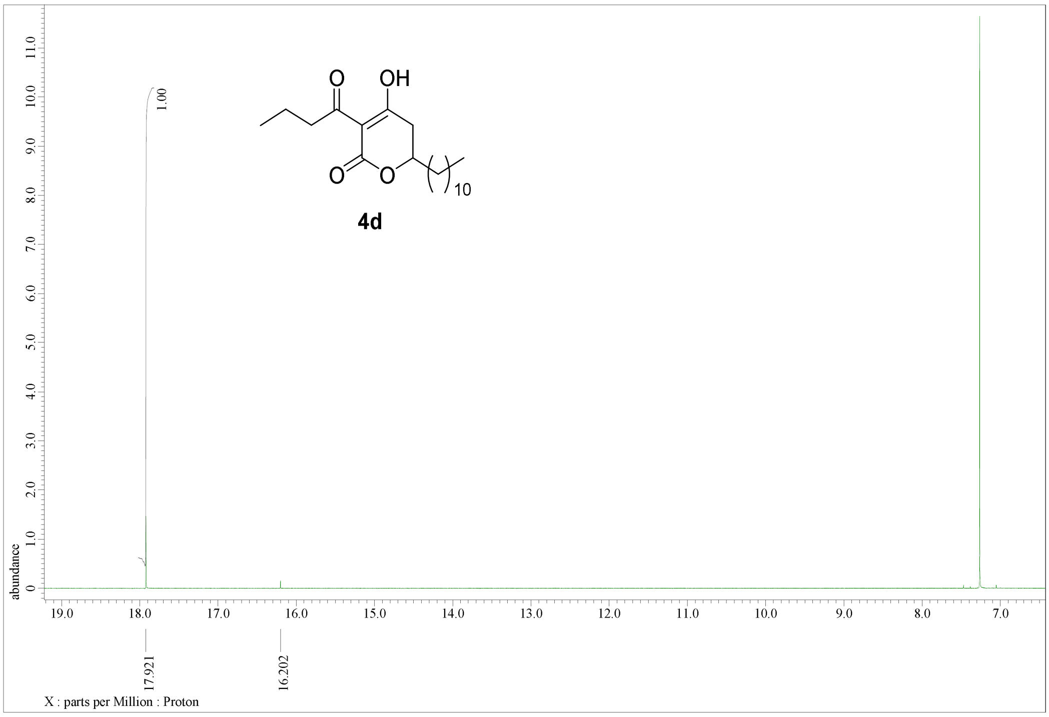

Figure S24. ^{1}H NMR (500 MHz, $CDCl_3$) Spectrum of the Compound 4d.

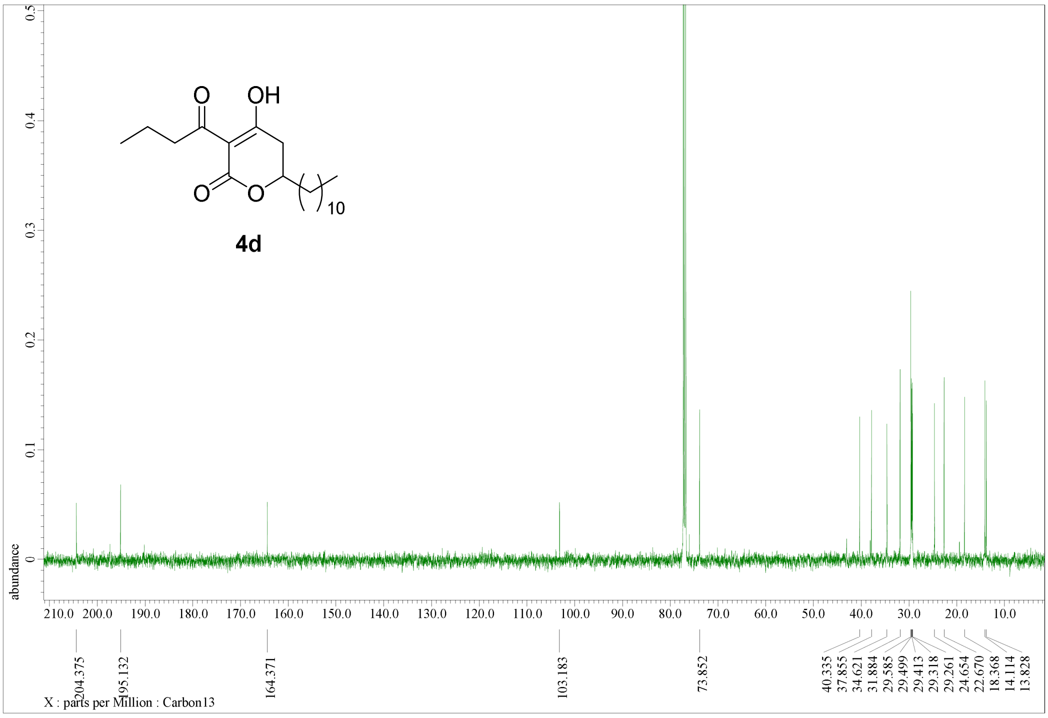

Figure S25. ^{13}C NMR (125 MHz, $CDCl_3$) Spectrum of the Compound 4d.

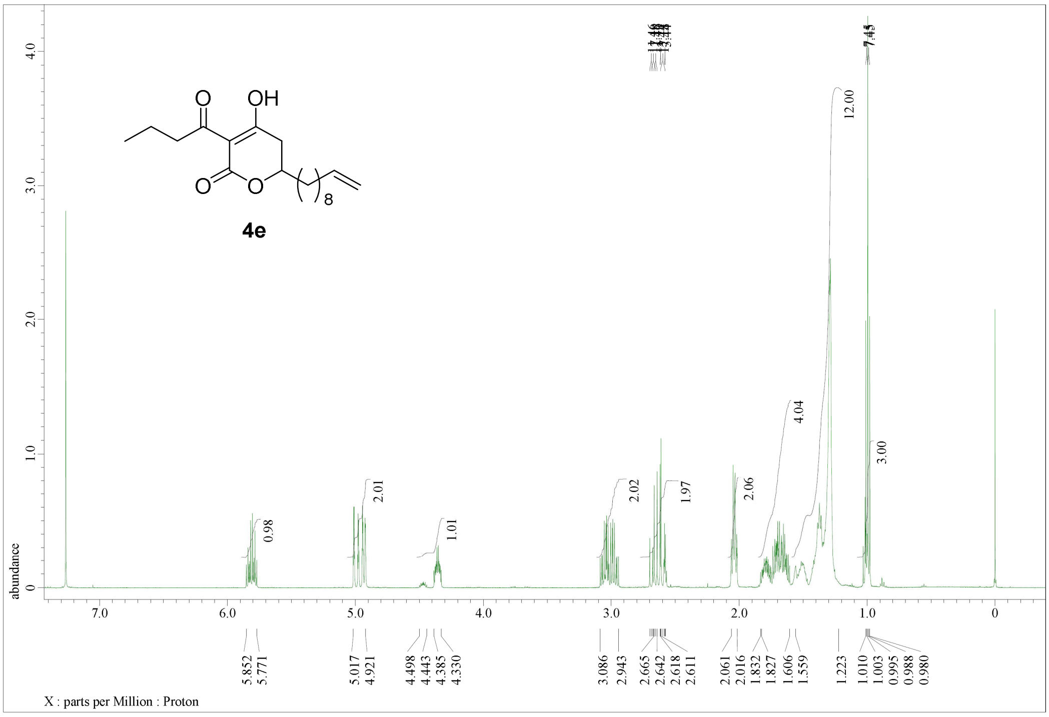

Figure S26. ^1H NMR (500 MHz, $CDCl_3$) Spectrum of the Compound 4e.

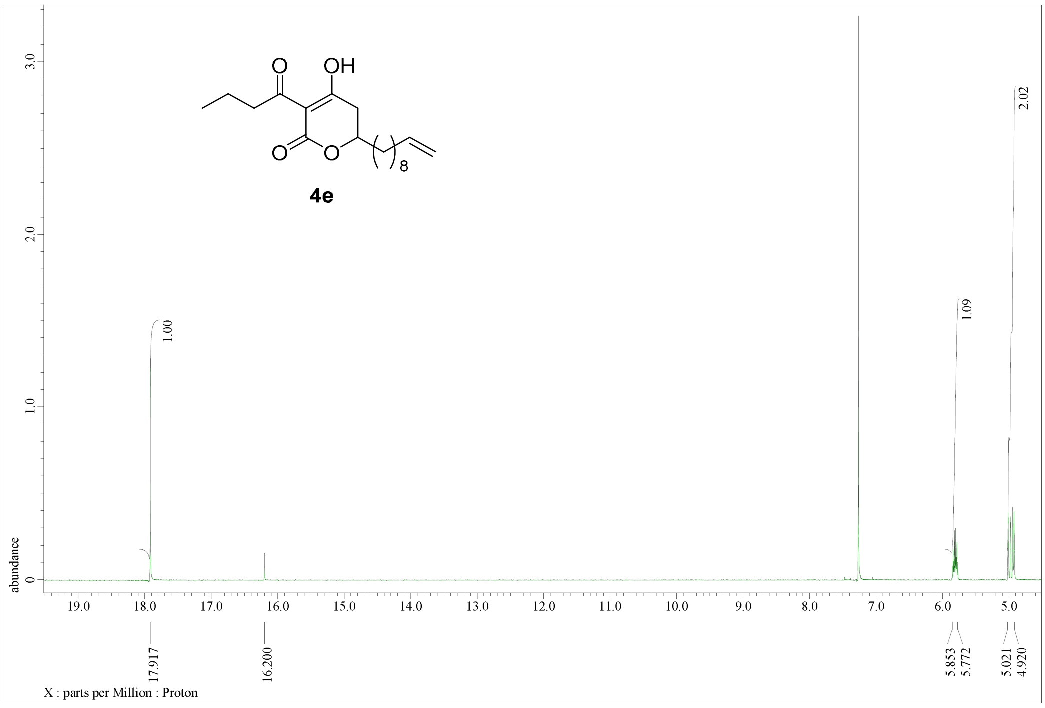

Figure S27. ^{1}H NMR (500 MHz, $CDCl_3$) Spectrum of the Compound 4e.

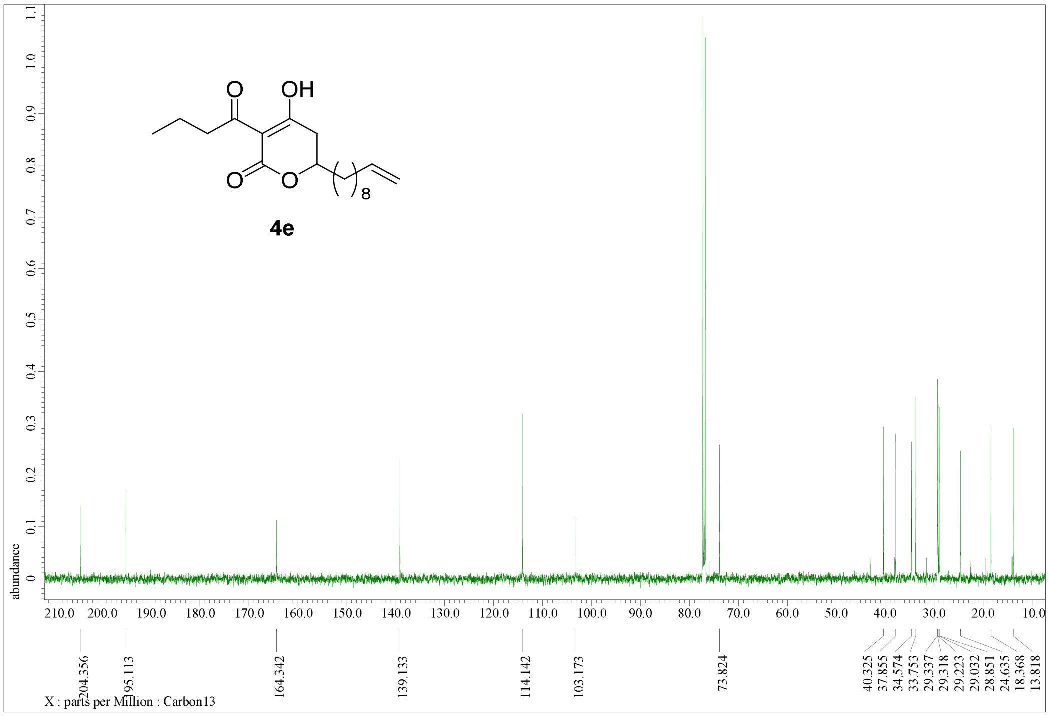

Figure S28. ^{13}C NMR (125 MHz, $CDCl_3$) Spectrum of the Compound 4e.

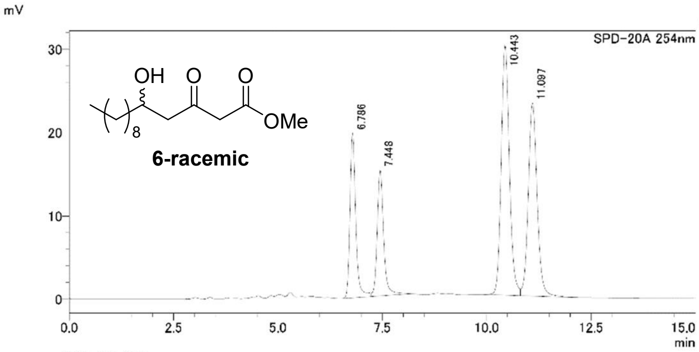

SPD-20A 254nm

peak	retention time	area	height	%
1	6.786	190524	19792	17.212
2	7.448	162026	15127	14.638
3	10.443	411416	30029	37.168
4	11.097	342934	23139	30.981

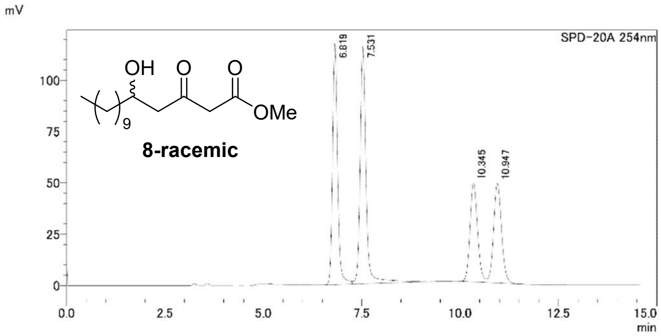

SPD-20A 254nm

peak	retention time	area	height	%
1	6.819	1049417	117440	29.115
2	7.531	1220643	115800	33.865
3	10.345	646055	48200	17.924
4	10.947	688286	48696	19.096

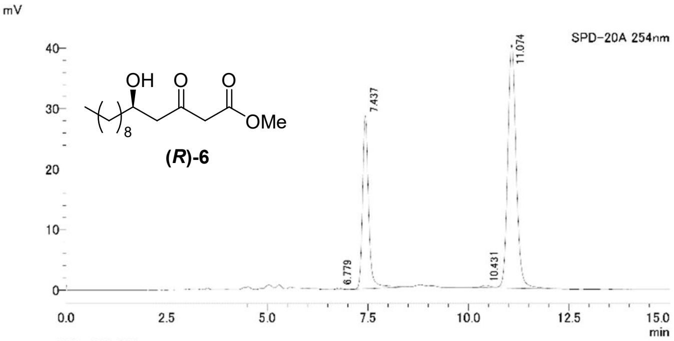

SPD-20A 254nm

peak	retention time	area	height	%
1	6.779	2156	235	0.239
2	7.437	300087	28623	33.324
3	10.431	6086	398	0.676
4	11.074	592197	40388	65.761

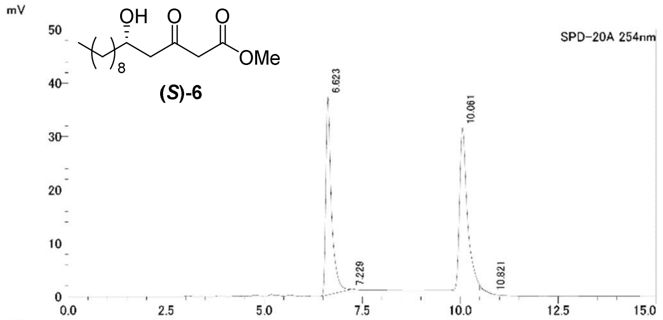

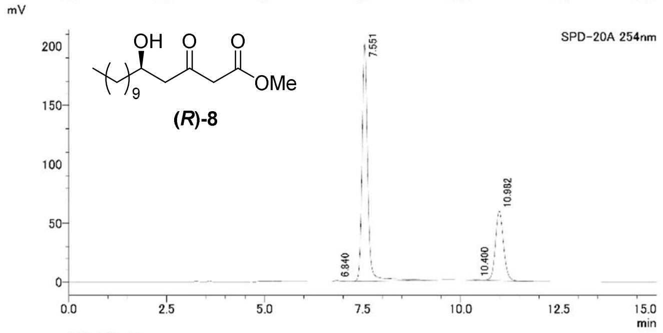

SPD-20A 254nm

peak	retention time	area	height	%
1	6.840	11441	1117	0.391
2	7.551	2053850	202048	70.235
3	10.400	6701	435	0.229
4	10.982	852257	59427	29.144

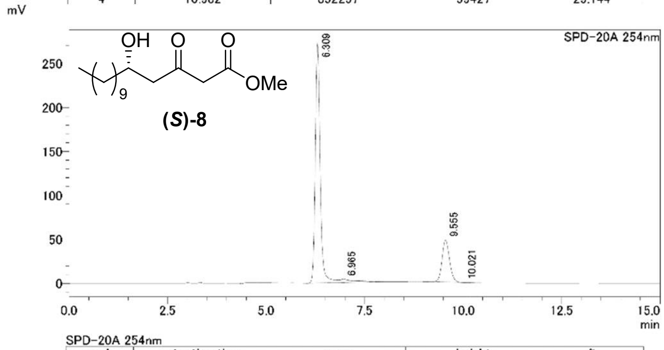

SPD-20A 254nm

peak	retention time	area	height	%
1	6.309	2437816	272551	78.902
2	6.965	17723	1540	0.574
3	9.555	632633	47807	20.476
4	10.021	1492	12	0.048

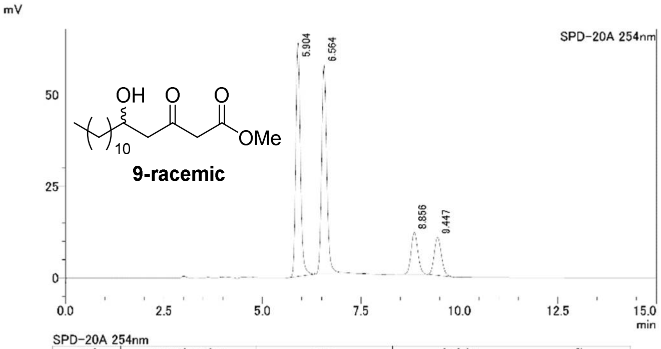

SPD-20A 254nm

peak	retention time	area	height	%
1	5.904	522582	63678	40.030
2	6.564	514718	56890	39.428
3	8.856	135731	11372	10.397
4	9.447	132444	10406	10.145

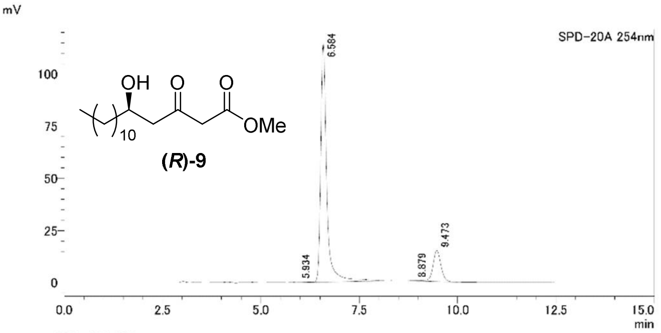

SPD-20A 254nm

peak	retention time	area	height	%
1	5.934	6393	364	0.444
2	6.584	1220870	114579	84.705
3	8.879	899	41	0.062
4	9.473	213154	14948	14.789

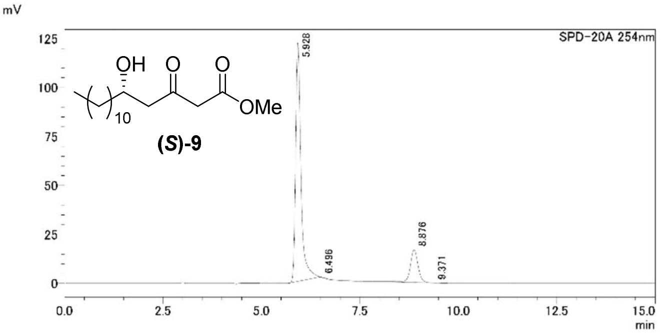

SPD-20A 254nm

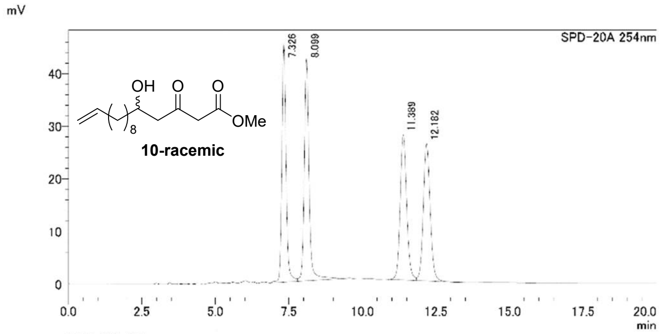

SPD-20A 254nm

peak	retention time	area	height	%
1	7.326	446261	45291	25.149
2	8.099	481879	42256	27.157
3	11.389	421550	27560	23.757
4	12.182	424759	26089	23.938

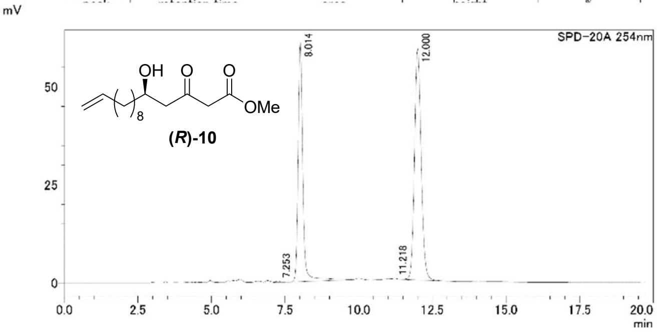

SPD-20A 254nm

peak	retention time	area	height	%
1	7.253	3551	324	0.219
2	8.014	684094	60883	42.145
3	11.218	1691	166	0.104
4	12.000	933857	59084	57.532

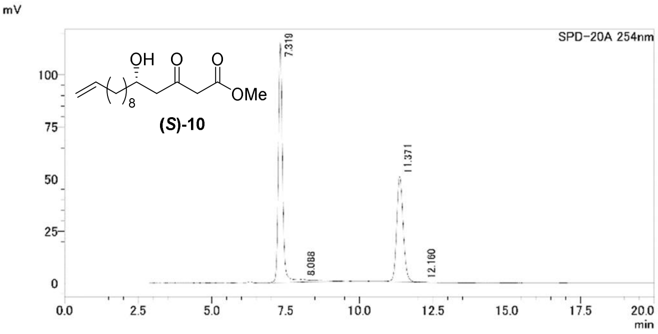

SPD-20A 254nm

peak	retention time	area	height	%
1	7.319	1181330	115021	60.559
2	8.088	3550	387	0.182
3	11.371	764274	50323	39.179
4	12.160	1566	132	0.080

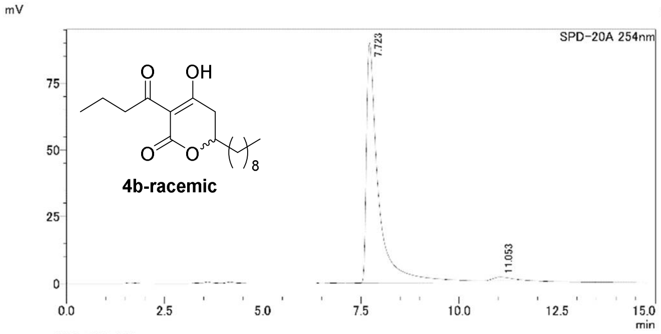

SPD-20A 254nm

peak	retention time	area	height	%
1	7.723	1970462	89967	97.534
2	11.053	49823	1389	2.466

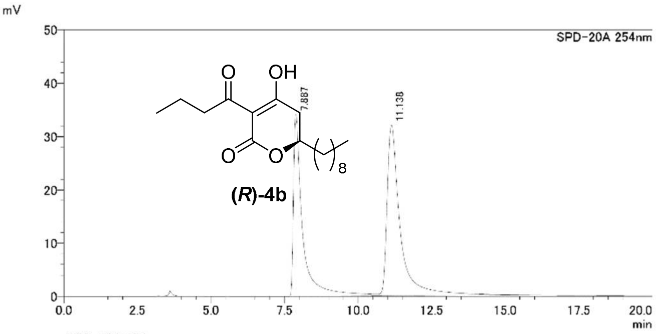

SPD-20A 254nm

peak	retention time	area	height	%
1	7.887	750592	34164	42.672
2	11.138	1008394	32080	57.328

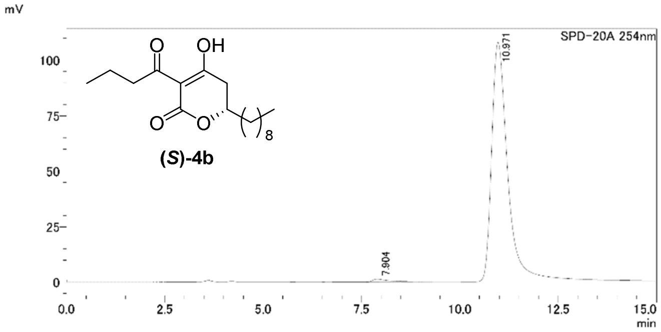

SPD-20A 254nm

peak	retention time	area	height	%
1	7.904	42295	1339	1.315
2	10.971	3174832	107876	98.685

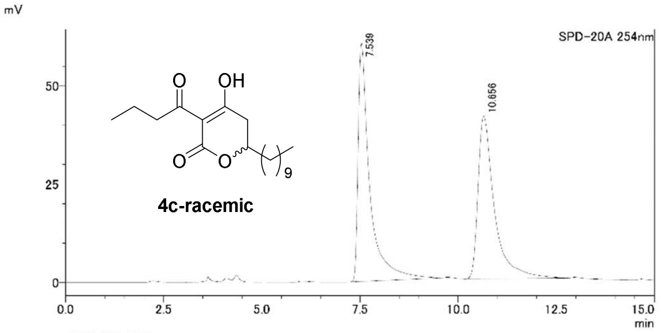

SPD-20A 254nm

peak	retention time	area	height	%
1	7.539	1259760	60730	51.334
2	10.656	1194287	41383	48.666

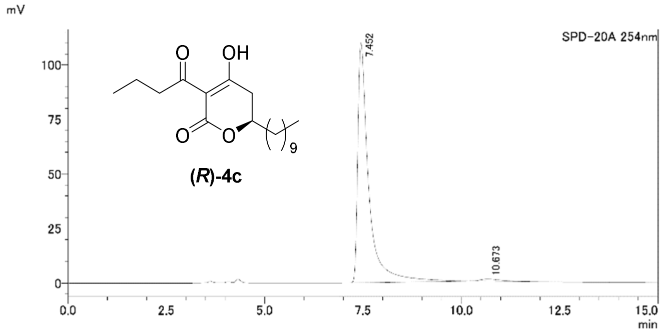

SPD-20A 254nm

peak	retention time	area	height	%
1	7.452	2150588	109825	98.473
2	10.673	33345	966	1.527

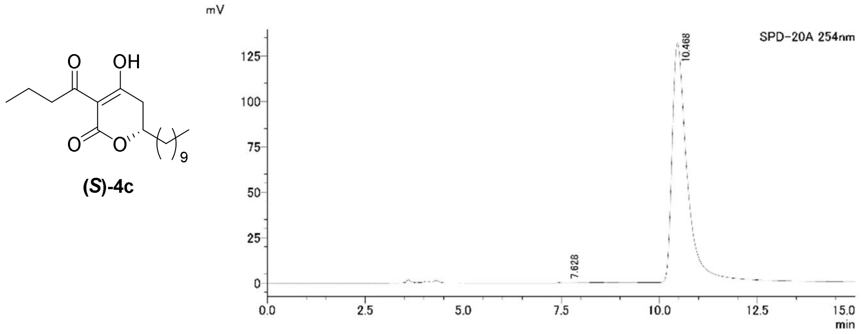

SPD-20A 254nm

peak	retention time	area	height	%
1	7.628	23849	548	0.645
2	10.468	3673167	131766	99.355
合計			132314	

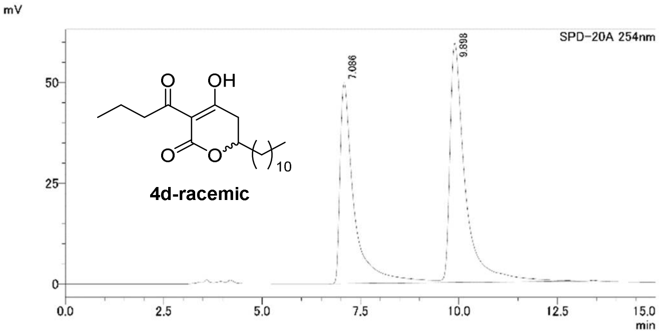

SPD-20A 254nm

peak	retention time	area	height	%
1	7.086	1258803	49855	43.691
2	9.898	1622316	59459	56.309

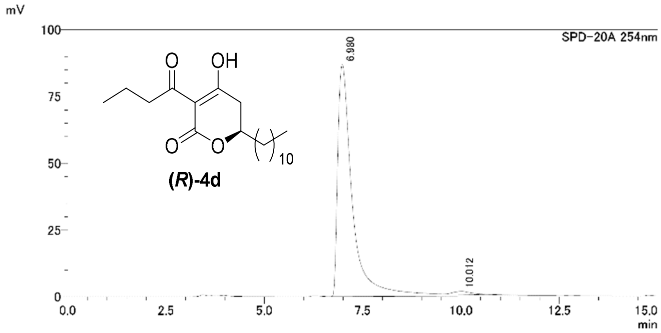

SPD-20A 254nm

peak	retention time	area	height	%
1	6.980	2334904	86933	98.354
2	10.012	39066	1154	1.646

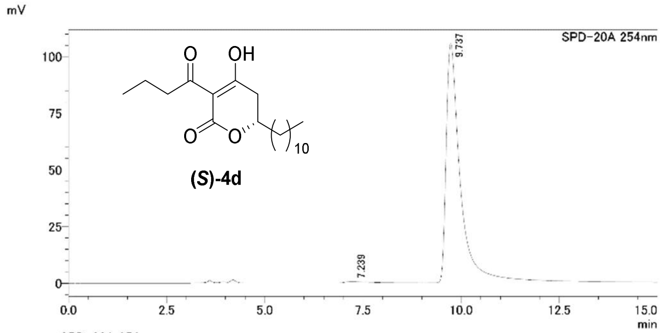

SPD-20A 254nm

peak	retention time	area	height	%
1	7.239	29993	626	1.077

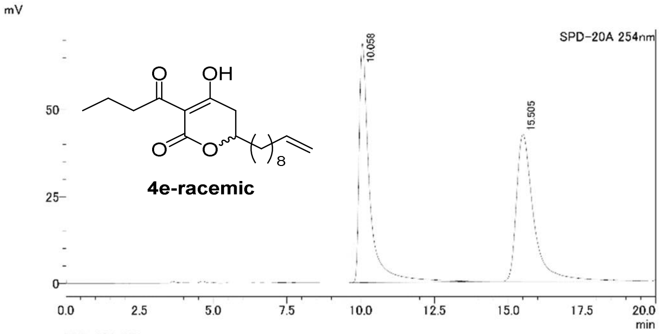

SPD-20A 254nm

peak	retention time	area	height	%
1	10.058	1721761	69009	49.776
2	15.505	1737225	42382	50.224

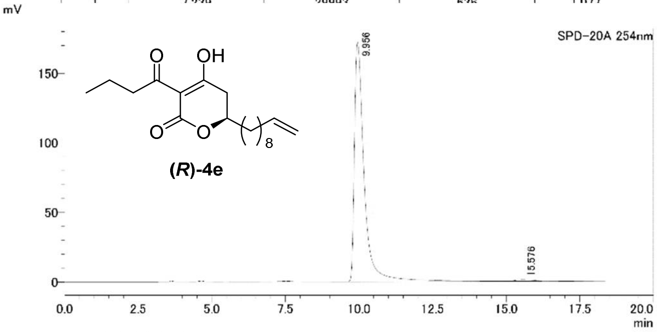

SPD-20A 254nm

peak	retention time	area	height	%
1	9.956	3981893	172135	98.519
2	15.576	59852	1149	1.481

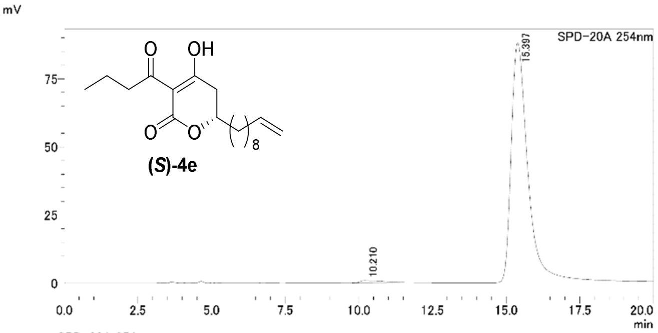

SPD-20A 254nm

peak	retention time	area	height	%
1	10.210	44661	816	1.247
2	15.397	3538106	87975	98.753
